# Spatial structuring of soil microbial communities in commercial apple orchards

**DOI:** 10.1016/j.apsoil.2018.05.015

**Published:** 2018-09

**Authors:** Greg Deakin, Emma L. Tilston, Julie Bennett, Tom Passey, Nicola Harrison, Felicidad Fernández-Fernández, Xiangming Xu

**Affiliations:** NIAB EMR, East Malling, West Malling, Kent ME19 6BJ, UK

**Keywords:** Distance-decay of similarity, Grass, Spatial autocorrelation, Trees

## Abstract

Characterising spatial microbial community structure is important to understand and explain the consequences of continuous plantation of one crop species on the performance of subsequent crops, especially where this leads to reduced growth vigour and crop yield. We investigated the spatial structure, specifically distance-decay of similarity, of soil bacterial and fungal communities in two long-established orchards with contrasting agronomic characteristics. A spatially explicit sampling strategy was used to collect soil from under recently grubbed rows of apple trees and under the grassed aisles. Amplicon-based metabarcoding technology was used to characterise the soil microbial communities. The results suggested that (1) most of the differences in soil microbial community structure were due to large-scale differences (i.e. between orchards), (2) within-orchard, small-scale (1–5 m) spatial variability was also present, but spatial relationships in microbial community structure differed between orchards and were not predictable, and (3) vegetation type (i.e. trees or grass and their associated management) can significantly alter the structure of soil microbial communities, affecting a large proportion of microbial groups. The discontinuous nature of soil microbial community structure in the tree stations and neighbouring grass aisles within an orchard illustrate the importance of vegetation type and allied weed and nutrient management on soil microbial community structure.

## Highlights

• Most variability in soil microbiota is between orchards (large scale).
• Within-orchard spatial structure of soil microbiota varies greatly between orchards.
• Different vegetation causes large differences in soil microbiota, particularly fungal community.

## 1 Introduction

Ecologists have long sought to understand the factors underlying the distribution and abundance of species. This is particularly true for the microorganisms in soil, where differences in soil chemistry give rise to large differences in the composition of soil microbial communities (Fierer and Jackson, 2006). Similarly, the type of overlying vegetation is important, with soil under trees supporting very different communities from those under grass (Rich et al., 2003). Until the early 1990s spatial variability in the distribution of soil organisms was often considered to be ‘random noise’, but slowly the interactions of a hierarchy of environmental factors, intrinsic population processes and disturbances are being described at scales ranging from millimetres to hundreds of metres (Ettema and Wardle, 2002). Improving our understanding of the factors that regulate the spatial distribution of soil biota in agroecosystems has relevance to many agronomic issues including nutrient cycling, the management of soil-borne plant pathogens and the efficacy of various microbial inoculants.

Two mechanisms are frequently proposed to account for differing spatial patterns of soil microbial diversity. The first is a more nuanced version of the generalisation that ‘…everything is everywhere, but the environment selects…’ (Baas Becking, 1934) and is predicated on the assumption that spatial patterns of microbial diversity are driven by environmental heterogeneity, rather than dispersal limitation. Thus, the composition of a given microbial community is dependent upon deterministic processes for the environmental filtering of component species, according to their ecological fitness for specific niches. Where deterministic processes operate, increasingly dissimilar microbial communities can be expected to develop under concomitantly different environmental conditions (Martiny et al., 2011); or along an environmental gradient (Green and Bohannan, 2006). The second mechanism is that dispersal processes determine the similarity of microbial communities. The physical structure of the soil matrix is assumed to limit migration of microorganisms and consequently, similar communities develop at neighbouring sites irrespective of any environmental differences. At small spatial scales, habitat heterogeneity declines and stochastic processes exert a greater influence on community composition than deterministic processes (Chase, 2014), but at larger spatial scales this can produce pronounced spatial structuring of microbial communities in soil (Ettema and Wardle, 2002).

One commonly reported spatial patterning of ecological communities is the negative relationship between community similarity and spatial distance, known as distance–decay of similarity (Nekola and White, 1999). Distance-decay of similarity is believed to result from a combination of deterministic processes (environmental filtering), and stochastic or neutral processes (dispersal limitation and ecological or evolutionary drift) (Bahram et al., 2015). Although environmental filtering is an obvious underlying mechanism for the emergence of distance-decay relationships, they can also arise where the environmental conditions are comparable, but there is significant drift (Bell, 2010). Other factors that influence the distance decay of community similarity include environmental periodicity, which, depending on the scale of observation relative to the periodicity, can either result in a lack of distance decay, or facilitate its detection.

Microorganisms are the most diverse and complex component of soil biodiversity and participate in many of the ecological interactions and processes required for the regulation of pests and diseases, water and nutrient retention, and maintenance of soil structure (Barrios, 2007). Managing soil biodiversity, and the community-level processes therein, has been highlighted as a major challenge in the transition to more sustainable agricultural systems (Lemanceau et al., 2015). It is therefore surprising that, in contrast with natural habitats, the distance-decay of similarity for microbial communities in cropped soils has received little attention. As a multi-layer assemblage of plants in a long-term complex spatial arrangement (Simon et al., 2017), commercial apple orchards are an interesting agroecosystem in which to study plant-soil-microbial interactions. The perennial nature of the plant communities in the rows of trees and the grass aisles means that soil management is constrained by time and space, and above- and below-ground ecosystems have greater stability than that associated with annual cropping. The low frequency of physical disturbance also means there is ample opportunity for the development of multiple bi-directional plant-soil-microbial interactions.

In this paper we report an investigation of the spatial similarity, specifically distance-decay, for microbial communities in soil under the two plant communities present in two long-established orchard sites with contrasting agronomic characteristics. We used a spatially explicit sampling strategy to collect soil from under recently grubbed rows of apple trees and under the grassed aisles. Culture-independent next generation sequencing techniques were used to profile soil microbial communities in soil. We hypothesized that (1) there would be coupling between vegetation composition and soil microbial assemblages, (2) the presence of environmental gradients would favour the development of distance-decay of similarity in soil microbial communities, and (3) distance-decay of similarity would be modified by management decisions such as the intensity of operations and tree spacing (i.e. environmental periodicity).

## 2 Materials and methods

### 2.1 Study design

Soil microbial communities were profiled in soil samples taken from two geographically and agronomically distinct apple **orchards**. Within each orchard, soils were sampled from two **vegetation types**: former tree stations and the adjacent grassed aisles; which were divided into three **blocks** of ca. 20 m long, each with eight consecutive trees (i.e. eight pairs of tree and aisles samples). The spatial location (i.e. the distance between sampling points within each orchard) was also recorded to enable calculation of spatial autocorrelation. At each sampling point (tree station or adjacent grassed aisle), three analytical replicate soil samples were taken.

### 2.2 Orchards

The two orchards sampled represented the contrasting rootstock and scion combinations typical for the production of dessert/culinary and cider apples (*Malus pumila* Miller) in the UK. The first orchard, planted with dessert/culinary varieties, was located in south-east England on Wickham series soil (Table 1). Between 1992 and the winter of 2014–15 this site was planted with ‘Bramley’s seedling’ and ‘Golden Delicious’ on ‘M.9’ rootstock. Prior to grubbing, the trees had been grown in north–south orientated rows 3.75 m apart, with an in-row tree spacing of 1.6 m. Groundcover in the aisles between the tree rows was mainly grasses (e.g. *Lolium perenne* L., *Poa pratensis* L., *Agrostis* spp. and *Festuca* spp.), with occasional weeds, which was kept short by periodic mowing during the growing season. The soil directly under the trees was kept mostly free of vegetation by regular application of herbicide.

**Table 1:** Selected geographical information and climate statistics for both sites.

	Dessert Orchard	Cider Orchard
*Geographical information*		
Latitude	51.210596	52.251020
Longitude	0.601664	-2.301711
Altitude (m a.s.l.)	80	65
Slope (° and orientation)	7, south-facing	2, south-facing
Soil type†	Eutric Luvic Planosol	Chromic Vertic Luvisol

*Regional climate statistics*‡		
Minimum monthly mean air temperature (°C)	1.4	0.9
Maximum monthly mean air temperature (°C)	22.6	21.6
Ambiental air temperature range (°C)	6.4–14.7	5.9–14.1
Minimum mean soil temperature, 10 cm (°C)	−1.9	−1.0
Maximum mean soil temperature, 10 cm (°C)	26.5	20.0
Ambiental soil temperature range, 10 cm (°C)	5.0–17.7	6.8–13.7
Minimum monthly mean soil temperature, 30 cm (°C)	0.7	No data
Maximum monthly mean soil temperature, 30 cm (°C)	24.4	No data
Ambiental soil temperature range, 30 cm (°C)	6.1–16.5	No data
Air frost (days)	47.5	49.3
Sunshine (hours)	1634	1554
Rainfall (mm)	673	665

† Soil nomenclature is in accordance with the recommendations of the IUSS Working Group WRB (2015).

‡ Regional climate statistics are mean values for the period 1981–2010 (2000–2015 for soil temperature), as measured by UK Meteorological Office weather stations within 10 km and 35 km of the dessert and cider orchards, respectively.

The second orchard was located in the West Midlands of England, approximately 230 km to the north-west of the dessert orchard, on Whimple series soil (Table 1). Since 1988 the orchard had been planted with cider apple varieties, the most recent planting prior to sampling was the cultivar ‘Katy’ on two different rootstocks: ‘M.M.106’ and ‘M.M.111’. The trees were planted in north–south orientated rows 5.5 m apart, with an in-row tree spacing of 2.75 m. The planting of ‘Katy’ was grubbed after 12 years in 2014. Before 2002, the site had been planted with the cider apple variety ‘Bulmer’s Norman’ on seedling rootstock at planting distances of 6.6 m between rows, and 2.6 m within rows. The former rows of the ‘Katy’ planting, sampled for this study, had been aisles in the ‘Bulmer’s Norman’ planting. There was no record of soil sterilization or the application of organic amendments to the land, either before or since 1988. A mixture of herbaceous plants (mainly grasses) was present in the aisles and a vegetation-free ‘herbicide strip’ was maintained under the rows as described above.

### 2.3 Soil sampling and analysis

The dessert orchard site was sampled on May 1, 2015. A single row of former tree stations running down the slope was split into three blocks, blocks were separated by a distance of 3 m and within each block there were eight paired sampling points – former tree stations and corresponding grass aisle positions, 1.5 m–1.7 m to the west.

The cider orchard was sampled on June 3, 2015. The rows at this site were too short to accommodate three blocks within a single row. Consequently, two blocks were in one row, separated by a 4.7 m gap and the third block was adjacent to the first, in the former row to the west. According to available records and knowledge, the former trees at the tree station points in blocks 1 and 2 had ‘M.M.106’ as a rootstock; with either ‘M.M.106’ or ‘M.M.111’ being the rootstock of the former trees at the tree station points in block 3. Once again, there were eight paired sampling points in each of the three blocks, with grass aisle positions being 2.75 m to the west of the tree station point.

Three replicate soil cores (2.5 cm diameter, containing soil from 5 cm to 20 cm depth) were taken circa 15 cm apart from each other at each type of sampling point (grass aisle and tree station). Each individual core was placed into a labelled polythene sample bag. Samples from the dessert orchard were taken the short distance (ca. 17 km) back to NIAB EMR at ambient temperature, while samples from the cider orchard were placed in an electric cool-box (ca. 5 °C–10 °C) for transportation (280 km) back to NIAB EMR. Samples were stored on arrival in a cold-store at 5 °C for ca. 48 h (cider) or 96 h (dessert) before subsamples of 0.25 g were taken from each soil core. Subsamples were placed in 2 ml micro-tubes and stored at −80 °C prior to DNA extraction.

Selected physico-chemical characteristics were determined on composite soil samples, consisting of 20 g aliquots from each core, for a given sampling point type within a block (i.e. 24 individual cores). Composite soil samples were homogenized by sieving to 2 mm in the field-moist state before analysis. Soil pH was measured in water (1:2.5 w/v), available K and Mg were quantified in 1 M ammonium nitrate extracts by atomic absorption spectroscopy and available P was quantified colorimetrically in 1 M bicarbonate extracts according to MAFF/ADAS (1986) by Natural Resource Management (NRM) Ltd, Bracknell, UK. Particle size distribution across three classes: sand (2.00–0.063 mm), silt (0.063–0.002 mm) and clay (<0.002 mm) were determined by laser diffraction (NRM Ltd), with subsequent texture attribution according to Avery (1973). Total organic carbon and total nitrogen contents were determined on oven-dry (105 °C), ball-milled subsamples by combustion in an elemental analyser (Quaternary Scientific (Quest), Reading, UK).

### 2.4 DNA extraction and sequencing

Total genomic DNA was isolated from 0.25 g of each soil sample using the PowerSoil DNA Isolation Kit (MoBio Laboratories) with minor modifications as described below. Before bead-beating in a FastPrep instrument (FP120, Bio 101, Thermo Savant, Qbiogene), the samples were incubated in lysis solution at 65 °C for 10 min., thereafter they were homogenised by two 20 s cycles of bead beating at a power setting of 5.0, with 5 min. on ice between cycles. The DNA released was further separated from soil components and purified according to the kit protocol. DNA was quantified using Nanodrop 1000 spectrophotometer (Thermo Scientific) and diluted to 2 ng µl^−1^.

Non-overlapping variable Internal Transcribed Spacer (ITS) regions of ITS1 and ITS2 were amplified using primers EkITS1F: (5′-CTT GGT CAT TTA GAG GAA GTA A-3′) and Ek28R: (5′-AT ATG CTT AAG TTC AGC GGG-3′). The V4 variable region of the 16S rRNA gene was amplified using primers F341 (5′-CCT ACG GGN GGC WGC AG-3′) and R805 (5′-GGA CTA CHV GGG TAT CTA ATC C-3′). The two primer sets were modified at the 5′ end with adaptors, TCG TCG GCA GCG TCA GAT GTG TATAAG AGA CAG – forward adaptor and GTC TCG TGG GCT CGG AGA TGT GTA TAA GAG ACA – reverse adaptor. ITS PCR conditions consisted of an initial denaturation at 95 °C for 5 min. followed by 30 cycles of: denaturation, 94 °C for 30 s, annealing, 52 °C for 60 s, elongation, 72 °C for 60 s, and a final elongation of 72 °C for 7 min. 16S RNA PCR conditions were as above, but for annealing the temperature was increased to 55 °C and the number of cycles reduced to 25. All PCR reactions were carried out with 1X PCR buffer minus Mg (Invitrogen, Life Technologies, USA), 2 mM MgCl_2_ (Invitrogen, Life Technologies, USA), 0.2 mM dNTP (Fisher Scientific), 0.2 mM forward and reverse primers each (Integrated DNA Technologies), 1U Platinum Taq DNA Polymerase (Invitrogen, Life Technologies, USA), and 8 ng template DNA. The reaction volume was made up to 25 µl with molecular biology reagent water (Sigma, UK).

PCR products were purified using a proprietary clean-up procedure (Agencourt AMPure XP PCR purification system, Agencourt Bioscience, Beverly, Massachusetts) in which PCR amplicons 100 bp and larger were selectively bound to paramagnetic beads and separated from contaminants by magnetism. Both fungal and bacterial amplicons originated from a single template, so were pooled corresponding to individual soil cores and were ligated to Illumina compatible adapters using a Nextera XT Index sample barcoding kit (Illumina). The quality of the final products was quantified by a Nanodrop 1000 spectrophotometer (Thermo Scientific) and a Qubit2.0 fluorometer (Life Technologies, USA). The samples were diluted to 10 ng µl^−1^ and pooled in a 4 nM library. The amplicon library was denatured using 1 mM NaOH and diluted to 17.5 pM as per the manufacturer’s protocol (Illumina 16S Metagenomic Sequencing Library Preparation protocol). The pooled library was sequenced using the 300 bp paired-end protocol on an Illumina MiSeq instrument running Miseq Reagent kit V3 (Illumina) (16 cycles indexing and 301 cycles, total 618 cycle run). The heterogeneity of the samples was then increased by combining (20% v/v) the diluted and denatured amplicon library with a denatured PhiX library of equimolar concentration.

### 2.5 Bioinformatic analysis of sequence reads

FASTQ reads were demultiplexed into bacterial (16S) and fungal (ITS) datasets based on their primer sequences. Reads with any ambiguous positions in the primer region, or non-matching forward and reverse primers were discarded. All operational taxonomic unit (OTU) processing was carried out with the UPARSE 9.0 OTU clustering pipeline (Edgar, 2013) unless specified otherwise.

As recommended in the UPARSE pipeline, sequences were processed in two steps in order to generate OTUs and to classify sequences into OTUs. Firstly, sequences were filtered with very stringent criteria to generate OTUs and to obtain a representative sequence for each OTU. Then to generate an OTU table (frequency of each OTU in each sample), all sequences were filtered with less stringent criteria and aligned with the OTU representative sequences for cluster analysis.

#### 2.5.1 Generating OTUs and assignment of taxonomic rank

16S forward and reverse reads were merged with a maximum difference in overlap of 5%. Merged reads containing adapter contamination, or fewer than 300 nucleotides (NT), were excluded from further analysis. Remaining merged reads were then filtered for quality with a maximum expected error threshold of 0.5 per sequence (Edgar and Flyvbjerg, 2015). Finally, forward and reverse primer sequences were removed.

The expected distance between ITS primers was larger than twice the MiSeq read length; consequently, forward (ITS1) and reverse (ITS2) reads could not be merged and were treated as unpaired reads. Unpaired reads which contained both forward and reverse primers, or the adapter sequences, were excluded from analysis. Reads shorter than 200 NT were discarded. Forward and reverse reads were quality filtered with an expected error threshold set to 1 per sequence. Ribosomal 18S and 5.8S regions were identified in ITS1 reads using HHMMER3 v 3.1b2 (Wheeler and Eddy, 2013) and hidden Markov models provided with ITSx (Bengtsson-Palme et al., 2013). To reduce type I errors, only reads containing both 18S and 5.8S regions were retained, other reads were discarded. To increase accuracy of taxonomic assignment, the 18S and 5.8S regions were then deleted (Bengtsson-Palme et al., 2013, Nilsson et al., 2011). Ribosomal 28S and 5.8S regions were identified in ITS2 reads. Again to reduce type I errors, only reads containing both regions were retained, other reads were discarded. The 28S and 5.8S regions were then deleted from the reverse reads. ITS1 reads and unique ITS2 reads (i.e. the paired ITS1 read had been discarded during pre-processing) were then combined for each sample.

When generating OTUs for either 16S or ITS datasets, all reads were padded (with Ns) so that they were all the same length. Sequences with fewer than 4 reads were discarded and all unique sequence reads were sorted by their respective frequencies. From these unique sequences, the UNOISE (Edgar and Flyvbjerg, 2015) algorithm (which also filters for chimeras) derived OTUs together with their representative sequences. The UTAX algorithm (http://drive5.com/usearch/manual/tax_conf.html) then assigned each OTU representative sequence to taxonomic ranks by alignment with the gene sequences in the reference databases ‘Unite V7’ (ITS) (Koljalg et al., 2013) and ‘RDP training set 15’ (16S) (Cole et al., 2014). The USEARCH 8.1 calc_distmx algorithm (Edgar, 2010) was used to calculate a distance matrix between OTU sequences, for 16S and ITS sequences separately.

#### 2.5.2 OTU table generation

For 16S, the original sequence reads were merged with a maximum difference in the overlap set to 15%, and the merged pairs were aligned to the OTU representative sequences. For ITS, the original unfiltered forward and reverse reads were aligned separately to the OTU representative sequences. In all clustering analyses the threshold similarity value was set to 97%.

### 2.6 Statistical analyses

All statistical analyses were carried out in R 3.2.0 (R Core Development Team, 2008). OTU counts were normalised for library size using the median-of-ratios method implemented in DESeq2 (Anders and Huber, 2010, Love et al., 2014), and where appropriate transformed using the DESeq2 variance stabilisation transformation (VST). The three samples taken from each sampling point were treated as analytical replicates and the data were pooled; for sampling points with fewer than three valid replicates (due to sequencing failures), the mean of available replicates was adjusted accordingly. OTUs with fewer than 6 normalised reads across all samples were excluded from further statistical analysis. Principal components (PCs) were calculated from VST transformed OTU counts. All analyses were carried out separately for ITS and 16S data.

#### 2.6.1 Spatial analysis

Analysis of variance (ANOVA) was carried out on the first two PCs to estimate the contribution to the observed variance in each of the selected PC scores by orchard (cider or dessert), vegetation type (tree station or grass aisle), spatial location of each sampling point within each orchard (there being two corresponding samples – one from tree station and the other from grass aisle, for each location), and the interaction between orchard and vegetation type. Distance-decay relationships (autocorrelation) over a distance of 17 m were calculated separately for tree or grass samples within each orchard as the Pearson correlation coefficients for PC1 and PC2 scores.

#### 2.6.2 Diversity indices

Alpha (α) diversity (Chao 1, Shannon and Simpson) diversity indices were calculated using the R Phyloseq 1.12.2 package (McMurdie and Holmes, 2013). Ranks of the alpha diversity indices were subjected to ANOVA with the significance determined by a permutation test using the R lmPerm v2.1.0 package (Wheeler, 2016) to assess the effect of orchard, vegetation type, location within each orchard, and the interaction between orchard and vegetation type (tree station or grass aisle). Beta (β) diversity was estimated from the distance matrix calculated from the appropriate OTU table. Neighbour joining trees were constructed from the distance matrices using the Ape 3.4 package for R (Paradis et al., 2004). The R GUniFrac 1.0 package (Chen et al., 2012) was used to calculate both unweighted and weighted (by OTU count) UniFrac distances between samples. Permutation multivariate analysis of variance (PERMANOVA), as implemented in R with Vegan 2.3-1 (Dixon, 2003), was carried out to assess the effect of orchard, vegetation type and their interaction on the UniFrac distances. Statistical significance was calculated in PERMANOVA using F-tests based on sequential sums of squares from 9999 permutations of the raw data.

#### 2.6.3 Differential OTU abundance

DESeq2 was used to detect OTUs with differential relative abundances in relation to vegetation type, orchards and their interactions. The new statistical fitting algorithm of DESeq2 was used to account for the variance heterogeneity often observed in sequence data. In this algorithm the negative binomial distribution is used as an error distribution when comparing the abundances of each OTU between groups of samples by generalised linear modelling. This method was shown to be superior to other methods (McMurdie and Holmes, 2014), including rarefying samples. Consequently, comparisons of relative OTU abundance are based on DESeq2 analysis of raw counts data and rarefaction was not performed. In addition, DESeq2 also implements an algorithm that automatically filters OTUs before differential abundance analysis based on several criteria, including variance in abundance across samples and overall abundance level. The fitted model was: spatial location within each orchard, vegetation type (grass vs tree), orchard (cider vs dessert), and the interaction between vegetation type and orchard. Thus spatial location effects within each orchard were first removed before assessing the effects of other factors. To correct for the false discovery rate associated with multiple testing, the Benjamini-Hochberg (BH) adjustment (Benjamini and Hochberg, 1995) was used within DESeq2. Statistical significance was determined at the 5% level (BH adjusted).

## 3 Results

### 3.1 Soil physico-chemical properties

The two sites had contrasting soil physico-chemical properties (Table 2). At the dessert site the soil had uniform clay loam texture, but at the cider site the soil had predominantly silty clay loam texture (blocks 1 and 3, adjacent to each other) with block 2 (situated below block 1) containing a proportionately smaller silt fraction resulting in clay loam texture. Soil pH was slightly alkaline at the dessert orchard, ranging between 7.9 and 8.1 and was medium to strongly acid at the cider orchard (pH 5.4–5.8). Allied to soil pH, the concentrations of available (extractable) P and K were more than two-times greater in the dessert orchard than those in the cider orchard. Concomitant with the application of fertiliser and allied inputs to the trees, rather than the grass aisles, the nutrient content of the soil from the tree rows was greater than that from the grass aisles, at both sites. Nitrogen was the exception to this pattern and at the dessert orchard the grass aisles contained 44% more nitrogen than the tree rows, the contrast between rows and aisles was less pronounced at the cider orchard. Consistent with its much darker colouration, the organic carbon content of soil at the dessert orchard was at least two times greater than at the cider orchard.

**Table 2:** Selected physico-chemical characteristics of the soil at both sites.

	pH (1:2.5 w/v H_2_O)	Total organic C mg g^−1^	Total N mg kg^−1^	Available P mg kg^−1^	Available K mg kg^−1^	Available Mg mg kg^−1^	Sand 2.00–0.063 mm % w/w	Silt 0.063–0.002 mm % w/w	Clay <0.002 mm % w/w	Textural Class† (UK)
*Dessert orchard, tree row*										
Block 1	8.1	46.5	2.0	47.0	741	98	35	34	31	CL
Block 2	8.1	36.1	1.7	51.4	706	101	37	31	32	CL
Block 3	7.9	37.2	1.8	56.4	749	111	36	31	33	CL

*Dessert orchard, aisle*										
Block 1	7.9	54.9	2.8	29.6	488	76	36	31	33	CL
Block 2	7.8	48.9	2.7	30.2	463	60	36	31	33	CL
Block 3	7.9	41.2	2.2	27.8	405	55	37	30	33	CL

*Cider orchard, tree row*										
Block 1	5.8	18.8	2.1	13.9	243	207	15	55	30	ZCL
Block 2	5.4	14.7	1.7	5.6	133	142	37	41	22	CL
Block 3	5.4	19.4	2.1	18.3	232	140	20	52	28	ZCL

*Cider orchard, aisle*										
Block 1	5.8	20.8	2.2	10.0	201	227	18	53	29	ZCL
Block 2	5.7	16.2	1.9	4.0	96	153	22	50	28	CL
Block 3	5.8	20.0	2.2	5.9	157	206	15	55	30	ZCL

*P values from two-way analysis of variance*										
Vegetation	0.681	0.116	0.011	<0.001	<0.001	0.825	0.467	0.676	0.226	
Orchard	<0.001	<0.001	0.243	<0.001	<0.001	<0.001	0.003	<0.001	0.006	
Interaction	0.034	0.241	0.053	0.011	0.003	0.055	0.415	0.342	0.614	

Values are based on a single homogenisation of 24 subsamples.

† CL = clay loam and ZCL = silty clay loam texture, based on the UK soil texture triangle (Avery, 1973).

### 3.2 Sequence quality and generation of OTUs

In the 286 soil samples taken from linear transects in the two apple orchards the number of sequence reads per sample ranged between fewer than 1000, to more than 200,000. The four samples with the lowest number of read counts were excluded from further analysis. The total number of reads divided fairly evenly between fungi and bacteria (Table 1 in Deakin et al. (2018)). Within these reads 2141 and 6410 unique OTU sets could be identified for fungi and bacteria, respectively. The majority of reads for bacteria (71%) and fungi (56%) could be aligned back to these OTUs. Within all sites the OTU sets were characterised by a few OTUs with a very high read count and a much larger proportion having lower counts. At least 80% of reads were captured in the most abundant 10% of OTUs in all cases (Fig. S1). However, a large majority of OTUs did have more than 5 aligned reads (Table 1 in Deakin et al. (2018)).

A large number of the OTUs could not be reliably classified taxonomically. More than 36% of fungal and 23% of bacterial OTUs were not classified at the phylum rank and only 17% of fungi and 15% of bacteria could be classified at the taxonomic rank of genus (Table 2 in Deakin et al. (2018)).

### 3.3 Diversity indices

**Fig. 1:**
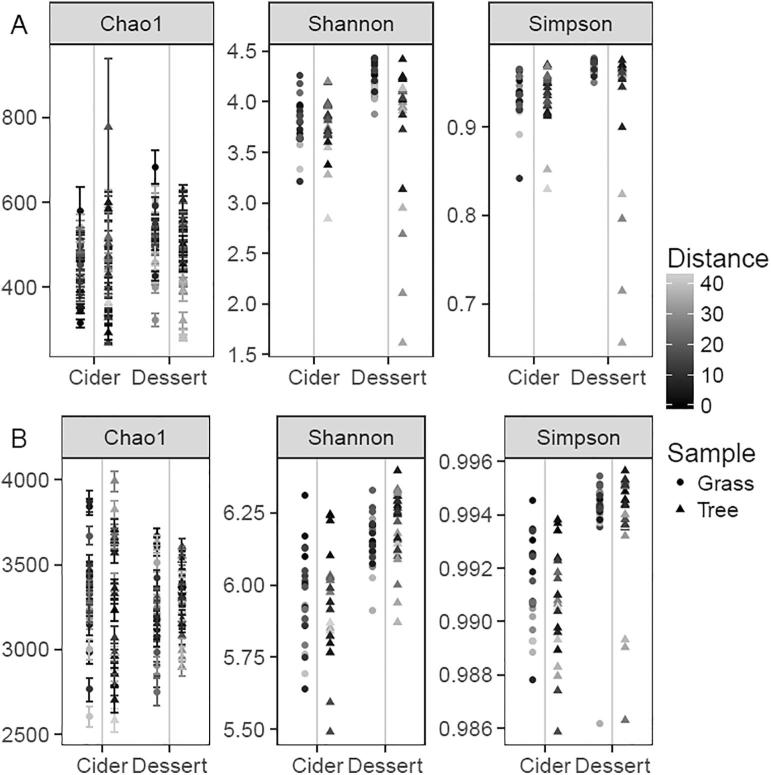
Alpha (α) diversity measures. Chao1, Shannon, Simpson alpha diversity measures for (A) fungal OTUs and (B) bacterial OTUs. The x-axes: C-G represents cider orchard grass aisle samples, C-T represents cider orchard tree station samples, D-G represents dessert apple orchard grass aisle samples and D-T represents dessert apple tree station samples.

Out of the three alpha diversity indices, Chao1 was least affected by experimental factors: circa 70% of total variability was unaccounted for (Table 3), but Shannon and Simpson indices suggested that there was greater fungal and bacterial diversity in the soil at the dessert orchard than at the cider orchard. Shannon and Simpson diversity indices also suggested that there were localised reductions in soil microbial diversity at some of the former tree stations relative to the corresponding grass aisle samples (Table 3, Fig. 1).

**Table 3:** Percentage of the variability in alpha and beta diversity indices accounted for by orchards, vegetation type (tree station vs grass aisle), spatial location within an orchard, and interaction between orchards and vegetation type.

Measure^†^	Vegetation type		Orchard		Location		Interaction		Residual
	%	*P value*	%	*P value*	%	*P value*	%	*P value*	
*Fungi*									
Chao1	0.17	0.351	9.82	0.005	18.23	0.981	0.38	0.725	71.39
Shannon	4.89	0.004	29.95	<0.001	14.53	0.442	5.64	0.003	44.99
Simpson	3.79	0.015	34.21	<0.001	16.81	0.177	4.34	0.031	40.85
UniFrac^‡^	5.34	<0.001	22.87	<0.001	18.27	0.109	4.08	<0.001	49.44
UniFrac^§^	8.88	<0.001	25.63	<0.001	15.84	0.109	7.73	<0.001	41.91

*Bacteria*									
Chao1	1.52	0.643	2.02	0.25	26.36	0.302	0.62	0.592	69.48
Shannon	0.11	0.961	38.66	<0.001	26.16	0.005	1.11	0.151	33.97
Simpson	1.19	0.226	45.88	<0.001	19.14	0.083	0.13	0.486	33.65
UniFrac^‡^	4.19	<0.001	36.75	<0.001	15.79	0.109	2.82	<0.001	40.46
UniFrac^§^	4.53	<0.001	58.78	<0.001	10.92	0.040	2.85	0.001	22.92

^†^Results from permutation ANOVA of ranks of α diversity indices (Chao1, Shannon and Simpson) or PERMANOVA (β diversity – UniFrac).

^‡^Unweighted UniFrac.

^§^Weighted UniFrac.

**Fig. 2:**
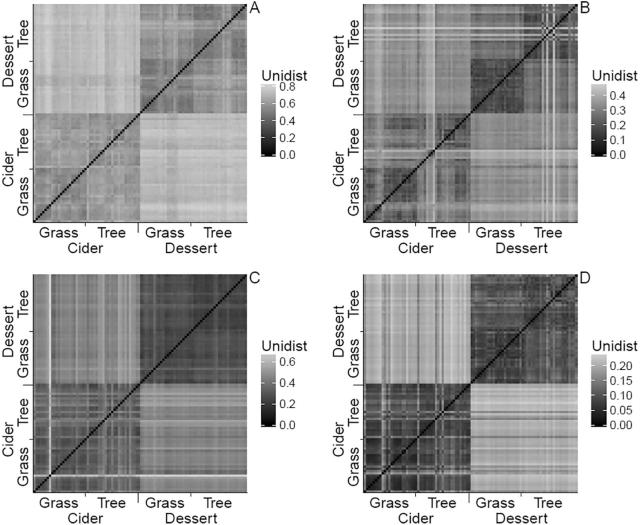
Unweighted (A: fungi, C: bacteria) and weighted (B: fungi, D: bacteria) UniFrac distance (β diversity indices - between samples calculated from a neighbour joining tree of phylogenetic distance between OTUs), illustrating between-orchard difference is much greater (darker in the heatmap) than within-orchard differences. The heatmaps have top left to bottom right diagonal symmetry, and samples have been ordered on both axis by the orchard and their physical location in each orchard.

UniFrac distances were used to visualise the large differences in diversity between-orchards, relative to the smaller differences within-orchard (i.e. beta diversity) (Fig. 2). Orchard differences explained a large proportion of the between-sample variance in the weighted (by OTU count) bacterial UniFrac distances - at least 23% and up to 59% (*p* ≤ 0.001, Table 3). For instance, the mean between-orchard and within-orchard differences in the weighted UniFrac distances are 0.20 and 0.10 (fungi), and 0.35 and 0.27 (bacteria), respectively. The corresponding values for the unweighted UniFrac distances are 0.47 and 0.32 (fungi), and 0.72 and 0.58 (bacteria).

### 3.4 Microbial community composition in tree and grass samples

Although similar numbers of OTUs were obtained from each orchard, bacterial OTUs were approximately three-times more numerous than fungal OTUs (Table 1 in Deakin et al. (2018)). For both fungal and bacterial datasets the majority of OTUs had low relative abundance (Fig. S1). The relative abundances of 341 fungal OTUs (Fig. S1A) and 1639 bacterial OTUs (Fig. S1D) were significantly (Benjamini-Hochberg adjusted *p* ≤ 0.05) different depending on vegetation type (tree station or grass aisle). Of these, 163 fungal OTUs and 632 bacterial OTUs were more abundant in the tree station samples than in the grass aisle samples. The relative abundance of a greater number of OTUs differed between the two orchards: 650 out of 2141 fungal OTUs (Fig. S1B) and 2105 out of 6410 bacterial OTUs (Fig. S1E), with fewer OTUs being associated with the interaction between orchard and vegetation type (Fig. S1CF).

OTUs from 31 classes in 8 fungal phyla and 88 classes in 32 bacterial phyla showed differential abundance between orchards and vegetation (Tables 3–6 in Deakin et al. (2018)). The fungal OTUs were associated with a range of ecological roles, predominantly saprotrophs. More specialist roles were associated with their respective hosts; the arbuscular-mycorrhizas (AM-fungi) were associated with the grass aisles (Glomeromycetes), whereas Tremellomycetes (‘jelly fungi’ – yeasts) that parasitise wood decay fungi were more abundant at tree stations. Other notable classes associated with the tree stations include the nematophagous Orbiliomycetes, Dothideomycetes which in addition to many saprotrophs is a class that contains *Venturia inaequalis* (Cooke) G. Winter (the causal agent of apple scab disease), Eurotiomycetes (wood decay saprotrophs) and Leotiomycetes (a diverse class which encompasses wood decay saprotrophs and the causal agent of brown rot of apple fruits *Monilinia fructigena* Honey). The bacterial community also contained many OTUs which are typically found in soil such as the low pH tolerant Acidobacteria and free-living heterotrophs such as Actinobacteria, Proteobacteria (which encompass the genus Pseudomonas) and Planctomycetes, which are indicative of wet conditions. Like the fungi, some classes of OTU were associated with a specific vegetation type, with Bacteroidetes, Chloroflexi, Firmicutes and Gemmatimonadetes being more abundant in the tree stations; while Verrucomicrobia were more abundant in the grass aisles.

Those OTUs where the difference in abundance between tree stations and grass aisles had a fold change value greater than 2.0 (significant at *p* ≤ 0.05, Benjamini-Hochberg adjusted) provided further detail on the composition of fungal and bacterial communities (Tables 7–8 in Deakin et al. (2018)). OTUs attributed to Microbotryomycetes and saprotrophic fungi, including *Monodictys* sp. and *Mortierella exigua* Linnem., were more abundant in the grass aisles than in the former tree stations; whereas the opposite is true for soil yeasts (*Cryptococcus aerius* (Saito) Nann., *Pichia* sp. and *Mrakia frigida* (Fell, Statzell, I.L. Hunter & Phaff) Y. Yamada & Komag.). Also associated with former tree stations were the Pyronemataceae, *Tetracladium* sp., the saprotroph and mycoparasite *Trichoderma* sp. and *Ilyonectria macrodidyma* (Halleen, Schroers & Crous) P. Chaverri & C. Salgado (anamorph of *Cylindrocarpon macrodidymum* Schroers, Halleen & Crous, a pathogen of woody plants). Within the bacterial community five groups of Acidobacteria, three classes of Proteobacteria, the Myxococcales (slime bacteria which degrade insoluble organic substances), *Terrimonas* sp. and Rhizobiales (symbiotic nitrogen fixers) were associated with the grass aisles. Symbiotic nitrogen-fixers and free-living chemoautotrophs in the family Xanthobacteraceae were associated with former tree stations, as were *Flavobacterium* spp., *Skermanella* sp., *Novosphingobium* sp. and *Pseudomonas* sp., a genus which contains known antagonists of soil-borne plant pathogenic fungi and plant growth promoting rhizobacteria.

**Fig. 3:**
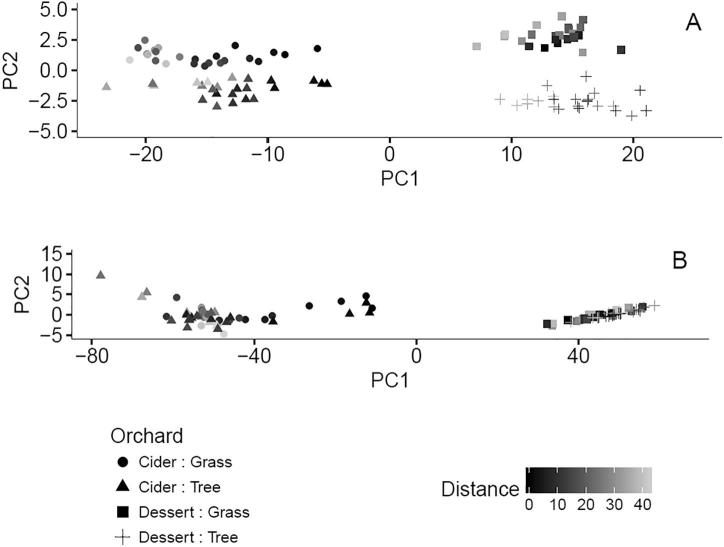
Principal component analyses of fungal (A) and bacterial (B) OTUs. For each Kingdom, principal components were calculated from the combined data from both orchards. Both graphs show PC1 vs PC2. Points represent orchard (cider or dessert) and vegetation type (tree station vs grass aisle). Point darkness represents the actual sample location along the sampling row direction within an orchard.

For both fungal and bacterial OTUs, principal components analysis revealed pronounced differences between and within the two orchards (Fig. 3). Principal component (PC) 1 separated the datasets according to orchard and accounted for 26% of the variation in the fungal OTU dataset and 50% of the variation in the bacterial OTU dataset. PC2 separated fungal OTUs by vegetation type, accounting for 7% of the variation in the dataset. There was no equivalent effect associated with PC2 for the bacterial OTU dataset: PC2 only accounted for 6% of the variation, most of which can be attributed to the spatial location of sampling points. However, if the effect of within-orchard spatial location is removed from PC1 and PC2 by ANOVA, then the latent effects of vegetation type can be detected at the dessert orchard (if the two orchards are considered separately) (Fig. S2A). The same statistical analysis of both the bacterial and fungal OTU datasets for the cider orchard resulted in tighter clustering of samples according to vegetation type (tree station or grass aisle) (Fig. S2BD, S3BD).

### 3.5 Distance-decay of similarity

**Fig. 4:**
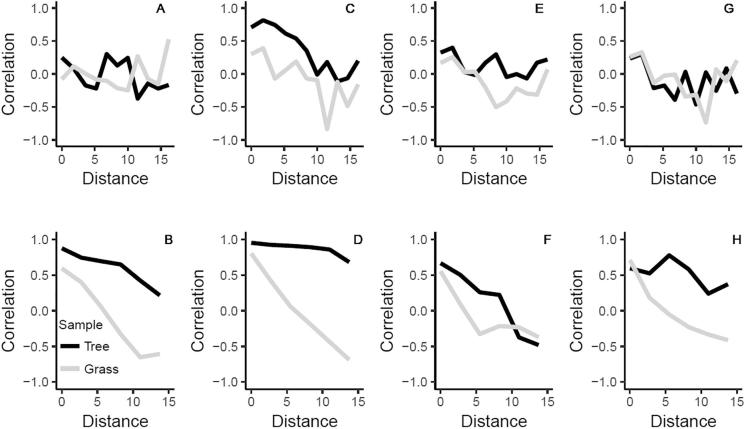
Autocorrelation of principal component (PC) scores for samples taken from tree stations (black lines) and grass aisles (grey lines). The x-axes show the spatial distance lags in metres and the y-axes the correlation for each distance lag. PC1 for *fungal* OTUs of the dessert (A) and cider (B) orchard; PC2 for *fungal* OTUs of the dessert (C) and cider (D) orchard; PC1 for *bacterial* OTUs of the dessert (E) and cider (F) orchard; PC2 for *bacterial* OTUs of the dessert (G) and cider (H) orchard.

Spatial autocorrelation analysis of PC1 and PC2 showed that the spatial pattern of soil microbial communities was inconsistent between the two orchards (Fig. 4). In the cider orchard, for the grass aisles in particular, soil microbial community structure was not homogeneous across the orchard (Fig. 4BDFH).

The fungal and bacterial soil microbial communities in the grass aisles at the cider orchard showed pronounced distance-decay of similarity, i.e. large-scale spatial structuring, in relation to both site-related (environmental) (Fig. 4BF) and plant-related (biotic) (Fig. 4DH) factors. For microbial communities associated with the former tree stations, the fungal component showed little spatial structuring amongst samples up to ∼10 m apart (Fig. 4BD), whereas the bacterial component showed strong site-related spatial structuring, but weak plant-related spatial structuring (Fig. 4FH). The spatial patterning of soil microbial communities associated with former tree stations was more obvious for PC1; whereas the inflexion points corresponding with 5.2 m in Fig. 4FG are suggestive of a small-scale effect of individual and neighbouring trees and it is notable that the tree planting distance was 2.6 m at this site.

At the dessert orchard large-scale patterns of spatial structuring were either not evident for bacteria (Fig. 4AEG) or were weak for fungi over a distance of 10 m (Fig. 4C). At the small-scale (<2 m), there is an indication of spatial similarity between soil microbial communities, which is consistent with a planting distance of 1.6 m for apple trees at the dessert orchard.

## 4 Discussion

Since the earliest days of soil microbial ecology, the diversity and functions of microbial communities in bulk soil and the rhizosphere have been demonstrated to be different (Hiltner, 1904). The application of next generation sequencing to the study of soil microbial communities continues to extend the results obtained from traditional culture- and microscopy-based techniques. The results of our study of samples obtained by systemic sampling of two orchards indicate that (1) most of the differences in soil microbial community structure in these cultivated soils were due to large scale differences (i.e. between orchards), (2) small-scale spatial variability was also present, but did not follow a predictable pattern, and (3) vegetation type (and associated agronomic practices) can significantly alter soil microbial community structure and affects a large proportion of microbial groups.

### 4.1 Large scale differences

Soil microbial community structure is a function of multiple plant-soil-microbial interactions operating at a range of spatial and temporal scales. Although the majority of factors do not operate independently of one another (Burns et al., 2015), there is a hierarchy in terms of their influence relative to each other. In the present study, the greatest determinant of soil microbial community composition was the orchard with which the community was associated, a result that is consistent with those reported by Peruzzi et al. (2017). Numerous studies have shown that the influence of edaphic factors on soil microbial communities is greater than the impacts of land use (Dequiedt et al., 2011, Kuramae et al., 2012), and vegetation community composition (Burns et al., 2015). The main factors driving microbial biomass and soil microbial community structure being pH, total organic carbon content (and allied properties such as cation exchange capacity and soil moisture content), phosphate content and soil texture (Dequiedt et al., 2011, Docherty et al., 2015, Kuramae et al., 2012). Distinct soil microbial communities develop in soils with contrasting physico-chemical properties because microbial populations show differential responses at all taxonomic ranks (Franklin and Mills, 2009). The bacterial component of soil microbial communities, for example, is well-known for being influenced by soil pH at the continental (Fierer and Jackson, 2006), landscape (Lauber et al., 2008), field (Franklin and Mills, 2003) and rhizoplane scales (Singh et al., 2008) probably because the pH ranges for optimal growth are narrower for bacteria than for fungi (Rousk et al., 2010).

There were pronounced differences in the physico-chemical properties of the soil at each orchard in the present study. Given the 2.5 unit difference in soil pH between the two orchards, it is therefore not surprising that the abundance of OTUs for pH-sensitive bacteria such as the Acidobacteria showed a particularly large difference between the two orchards. Furthermore, the Acidobacteria are oligotrophic bacteria and are less abundant in organic carbon-rich soils, whereas the Bacteroidetes and β-Proteobacteria are more abundant in organic carbon-rich habitats (copiotrophic bacteria) (Fierer et al., 2007), and OTUs in these taxonomic ranks also showed large orchard-specific differences.

### 4.2 Effect of plant host on microbial community structure in the rhizosphere

After soil physico-chemical properties, the most important factor for determining the composition of soil microbial communities is the vegetation community with which the sample was associated, our finding agrees with those of St. Laurent et al., 2008, Qin et al., 2016. Distinctive rhizosphere microbial communities develop in association with plants of different species (Burns et al., 2015, Haichar et al., 2008), and in our study, different soil microbial communities were detected at the former tree stations and in the grass aisles.

Vegetation type exerted a greater effect on the fungal component of the soil microbial communities in the dessert orchard than in the cider orchard, and differential abundances of various OTUs often reflected the ecological roles associated with specific taxonomic ranks. Fungi that form relationships with other organisms, such as obligate biotrophs, typically show greater habitat specificity than free-living organisms (Bahram et al., 2015), and there were multiple instances of such relationships within the orchards. Tremellomycete soil yeasts were associated with the former tree stations and members of this taxon not only parasitise wood-decay fungi, but also decompose ligno-cellulose and possibly pectic substances (Nakagawa et al., 2004, Stursová et al., 2012). OTUs representing the soil yeasts *Cryptococcus aerius* (Saito) Nann., *Mrakia frigida* (Fell, Statzell, I.L. Hunter & Phaff) Y. Yamada & Komag and *Pichia* sp. were more abundant in the former tree stations; the latter was previously identified as a common yeast in a cider orchard in the West of England (Beech, 1993). The nematophagous Orbiliomycetes were also associated with former tree stations and Mazzola et al. (2015) have previously reported the presence of nematophagous fungi in orchard soils. Other fungal taxa present in the orchards we studied and those studied by Mazzola et al. (2015) include the Eurotiomycetes, Pezizomycete, Leotiomycetes and Dothideomycetes. These taxonomic ranks include many wood-decay saprotrophs (Lundell et al., 2014) and fruit pathogens, e.g. *Venturia inaequalis* (Cooke) G. Winter (Dothideomycetes), the causal agent of apple scab, and *Monilinia fructigena* Honey (a Leotiomycete) which causes brown fruit rot. The Pyronemataceae (Pezizomycetes) are often considered to be ectomycorrhizas, but given that apple is an AM-fungal host (Miller et al., 1985) their presence in this ecological role seems unlikely. It is more likely that the representatives of this taxonomic rank are living as parasites or saprotrophs, or have formed symbiotic relationships with the abundant growth of bryophytes in the herbicide strips under the trees (Hansen et al., 2013). Other notable fungal OTUs associated with the former tree stations include the aquatic hyphomycete *Tetracladium* sp., which is increasingly being reported as a root endophyte (Sati and Arya, 2010), the saprotroph and mycoparasite *Trichoderma* sp. and *Ilyonectria macrodidyma* (Halleen, Schroers & Crous) P. Chaverri & C. Salgado (anamorph of *Cylindrocarpon macrodidymum* Schroers, Halleen & Crous). *Ilyonectria macrodidyma* is a pathogen of woody plants, frequently found in soil samples from tree rows and not the grass aisles (Kelderer et al., 2012). It has also been implicated in the development of apple replant disease (Tewoldemedhin et al., 2011).

Amongst the bacteria, as reported by many other studies of soil microbial communities, the most dominant taxonomic ranks were the Bacteroidetes, Chloroflexi, Firmicutes, Gammaproteobacteria (e.g. *Pseudomonas* sp.) and Gemmatimonadetes (Janssen, 2006). In common with the prevalence of saprotrophy within the fungal community, the Bacteroidetes are litter and soil dwelling bacteria, able to degrade cellulose (Stursová et al., 2012), i.e. copiotrophs in the ecological classification of Fierer et al. (2007). *Pseudomonas* sp. are renowned biocontrol agents, previously reported to be more abundant in tree station samples than in grass aisle samples from apple orchards in New York state (St. Laurent et al., 2010, Yao et al., 2006).

Saprotrophs also dominated the soil microbial communities found in the grass aisles, but the composition of this subset differed from that found at the former tree stations. It included both generalist and more specialist fungi, but notably not those able to degrade wood; reflecting functional adaptation of the soil microbial community to debris inputs from grasses and herbaceous species of plants rather than trees. *Monodictys* sp. is a generalist and was amongst the fungi commonly isolated from the standing senescent culms of graminids by Wong and Hyde (2001). Similarly, *Mortierella exigua* Linnem. is able to degrade cellulose and was described as an ‘…occasional decomposer fungus of grasslands…’ (Deacon et al., 2006). In agreement with the soil microbial communities of alpine grassland profiled by Mouhamadou et al. (2011), the soil microbial communities of the grass aisles contained AM-fungi (Glomeromycetes) and endophytes. Although Microbotryomycetes encompasses the ‘smut’ pathogens of graminids, it also includes yeasts in the plant pathogenic lineage Microbotryales. Like the Tremellomycetes at the former tree stations, Microbotryales yeasts are often mycoparasites (Begerow et al., 2017).

The dominant members of the bacterial community also reflected the greater abundance of cellulose in plant litter inputs. Flavobacteria are ubiquitous in soil, but are particularly abundant in habitats associated with plants such as the rhizosphere and phyllosphere. In comparison with their aquatic counterparts, terrestrial Flavobacteria have a greater abundance and diversity of genes for enzymes involved in the decomposition of plant cell wall carbohydrates such as xylose, arabinose, rhamnogalacturonan and pectin (Kolton et al., 2013). Similarly, the Verrucomicrobia and Proteobacteria are common soil and litter dwelling bacteria able to degrade cellulose (Stursová et al., 2012). Other notable OTUs include those representing *Novosphingobium* and *Pseudomonas* spp., genera which are considered to be beneficial and have been reported as contributing to reduced abundance of soil-borne plant pathogens in apple orchards (Caputo et al., 2015). The presence of symbiotic nitrogen-fixing Rhizobiales in soil from the grass aisles is also consistent with the widespread patches of *Trifolium repens* L. in the sward.

Continuous plantation of apple in the same area leads to reduced growth vigour and subsequent crop losses, i.e. apple replant disease (ARD) syndrome. The present results highlight the difficulties of making inferences about the candidate causal organisms of ARD via comparisons of microbial communities in replant with those at non-replant sites, including large plots in the same area or orchard. Such comparisons frequently lead to identification of a large number of microbial groups whose abundance differs between replant and non-replant sites. Restricting the comparisons made to between tree rows and grass aisles, in the belief that replant disease is more likely to occur in the original tree stations than in the grass aisles, does not provide further insight because the long-term presence of either trees or grasses will have resulted in numerous differences in many microbial groups in the soil. Thus, a better sampling scheme is needed for the identification of candidate causal organisms of ARD in which small-scale spatial variability is controlled. This type of sampling has been successful in identifying candidate causal microbes for reduced strawberry growth (Wei et al., 2016).

### 4.3 Spatial structure

The arrangement of apparently homogeneous rows of clonally propagated trees separated by grass aisles, which is characteristic of commercial apple orchards, confers considerable connectivity aboveground, facilitating the dispersal of many macro-organisms (Blaauw and Isaacs, 2014). Belowground the dispersal of microorganisms is constrained by the small size of microorganisms, relative to the physical soil matrix in which they live, and by the reliance on passive mechanisms of dispersal (Bahram et al., 2015). Thus, the distribution of soil microorganisms is intrinsically aggregated and frequently shows strong spatial structuring (Ettema and Wardle, 2002). Stochastic processes exert a greater influence on community assembly at small scales, than the deterministic processes arising from climatic and environmental gradients at large scale (Bahram et al., 2015).

At the dessert orchard, despite the presence of slope-related gradients in soil physico-chemical properties (e.g. pH, organic carbon content and clay content), the structure of soil microbial communities showed considerable heterogeneity across the spatial scales studied and there was no consistent evidence of distance-decay of similarity associated with measured edaphic factors. The fungal communities at the former tree stations, however, did show spatial correlation corresponding to the spacing of the trees (1.6 m). This suggests that the specialist fungi that form associations with trees and their debris are more abundant at the former tree stations and is consistent with the findings of Bahram et al. (2015). The spatial scale at which autocorrelation emerges depends not only on the size of host plants, but also the size of microorganisms themselves. In ecosystems that experience little macro-scale physical disturbance of soil the vegetative mycelium of an individual fungus can occupy tens of metres (Douhan et al., 2011), so depending on the sampling distances the same individual could be detected in multiple samples. In contrast, autocorrelation for bacteria can often only be detected at the millimetre scale (Zhou et al., 2004) which is a spatial scale several orders of magnitude smaller than the distance between our neighbouring samples.

The steep distance-decay of similarity curves for the soil microbial communities of the grass aisles at the cider orchard in the present study are typical for communities where dominant species are highly aggregated (Morlon et al., 2008), rather than communities with high spatial turnover as originally postulated by Harte et al. (1999). Strong environmental and climatic gradients are also often associated with steep distance-decay of similarity curves. Although the cider orchard lacked the slope-related gradients in soil physico-chemical characteristics present at the dessert orchard, these variables (i.e. pH, P, K and Mg) and soil texture were inherently more variable across the sampling area. Other processes that contribute to strong distance-decay of similarity include dispersal limitation and ecological drift. Wide distribution ranges, increased dispersal and migration rates, coupled with superior establishment success, environmental competence and hence persistence, as well as the action of drift in the absence of other environmental filters have a homogenizing influence and lead to weaker distance-decay of similarity patterns (Vellend, 2010). In cropped soils, long histories of extensive tillage operations and monoculture cropping also exert homogenizing influences (Buckley and Schmidt, 2001, Ettema and Wardle, 2002, Naveed et al., 2016). Conversely, the infrequency with which the soils in orchards are tilled is likely to increase heterogeneity.

In comparison with the soils subjected to conventional agricultural management practices, the microbial biodiversity of those used for less intensive organic farming often has greater species richness and decreased evenness (Hartmann et al., 2015). It is therefore surprising that the alpha diversity at the cider orchard was generally smaller than at the dessert orchard. Typically, cider orchards are managed less intensively than dessert apple orchards, where the requirements for fruit quality are much more stringent than those for apples destined for cider production. The slightly lower alpha diversity in the cider orchard soils is also at variance with the generalisation that richer and more diverse soil bacterial communities are found in neutral soils, relative to those in acidic soils (Fierer and Jackson, 2006). Climate and soil texture are lesser influences than soil pH (or organic carbon content) in the hierarchy of biotic and abiotic factors that can influence soil bacterial communities (Fierer, 2017). This suggests that management practices such as herbicide usage and fertilisation are exerting a significant influence on the structuring of soil microbial communities, possibly at different spatial scales from other environmental factors. The annual applications of herbicides to the tree rows, for example, could affect soil microbiota indirectly by killing opportunistic plants, and the transition from predominantly labile sources of organic inputs (i.e. root exudates) to more recalcitrant sources (i.e. dead plant biomass) would be likely to have an analogous effect on the microbial community (Tilston et al., 2014). It should, however, be noted that this difference may also result from the inherent differences between the two sites rather than different cropping histories.

Although there is an abrupt change in the species diversity and physiognomy of the overlying vegetation between the tree rows and the grass aisles aboveground, belowground the transition zone is likely to be wider owing to limited extension of tree roots into the soil beneath the grass aisles. Further quantitative and qualitative characterization of the transition zone and movement of soil microorganisms between tree rows and the grass aisles would yield useful information on the dispersal of soil microorganisms and mesofauna, such as free-living root lesion nematodes, in relation to integrated pest management and other cultural approaches to disease management. For example, increasing the botanical diversity of the grassed aisles is an emerging recommendation to increase the aboveground dispersal of predators and pollinators through orchards, but the belowground impacts on soil microbial diversity, soil health and the persistence of mycorrhizal inoculants and introduced biological control agents have yet to be characterised.

## 5 Conclusion

The results presented show that most of the differences in soil microbial community structure were due to large-scale differences (i.e. between orchards). Although within-orchard small-scale (1–5 m) spatial variability was present, such variability differed between orchards and did not have a predictable pattern. Within an orchard, vegetation type exerts a strong influence on a large number of microbial groups and results in significant differences in the structure of the soil microbial communities that develop under the rows of trees and in the grass aisles. The discontinuity in soil microbial community composition between the rows of trees and the grass aisles indicates the importance of orchard management; not only in terms of weed and nutrient management, but also for specific microbial groups.

## Conflict of interest

The authors declare no conflicts of interests.

## Appendix A Supplementary data

Supplementary data 1 Supplementary material.

Supplementary data 2 Data profile.

## References

[b0005] Anders S., Huber W. (2010). Differential expression analysis for sequence count data. Genome Biol..

[b0010] Avery B.W. (1973). Soil classification in the soil survey of England and Wales. J. Soil Sci..

[b0015] Baas Becking L.G.M. (1934). Geobiologie of inleiding tot de milieukunde.

[b0020] Bahram M., Kohout P., Anslan S., Harend H., Abarenkov K., Tedersoo L. (2015). Stochastic distribution of small soil eukaryotes resulting from high dispersal and drift in a local environment. Isme J..

[b0025] Barrios E. (2007). Soil biota, ecosystem services and land productivity. Ecol. Econ..

[b0030] Beech F., Rose A.H., Harrison J.S. (1993). Yeasts in cider-making. The Yeasts, Volume 5: Yeast Technology.

[b0035] Begerow D., Kemler M., Feige A., Yurkov A., Buzzini P., Lachance M.A., Yurkov A. (2017). Parasitism in yeasts. Yeasts in Natural Ecosystems: Ecology.

[b0040] Bell T. (2010). Experimental tests of the bacterial distance–decay relationship. ISME J..

[b0045] Bengtsson-Palme J., Ryberg M., Hartmann M., Branco S., Wang Z., Godhe A., De Wit P., Sánchez-García M., Ebersberger I., de Sousa F., Amend A., Jumpponen A., Unterseher M., Kristiansson E., Abarenkov K., Bertrand Y.J.K., Sanli K., Eriksson K.M., Vik U., Veldre V., Nilsson R.H. (2013). Improved software detection and extraction of ITS1 and ITS2 from ribosomal ITS sequences of fungi and other eukaryotes for analysis of environmental sequencing data. Methods Ecol. Evol..

[b0050] Benjamini Y., Hochberg Y. (1995). Controlling the false discovery rate: a practical and powerful approach to multiple testing. J. R. Stat. Soc. Ser. B.

[b0055] Blaauw B.R., Isaacs R. (2014). Flower plantings increase wild bee abundance and the pollination services provided to a pollination-dependent crop. J. Appl. Ecol..

[b0060] Buckley D.H., Schmidt T.M. (2001). The structure of microbial communities in soil and the lasting impact of cultivation. Microb. Ecol..

[b0065] Burns J.H., Anacker B.L., Strauss S.Y., Burke D.J. (2015). Soil microbial community variation correlates most strongly with plant species identity, followed by soil chemistry, spatial location and plant genus. AoB Plants.

[b0070] Caputo F., Nicoletti F., De Luca Picione F., Manici L.M.M. (2015). Rhizospheric changes of fungal and bacterial communities in relation to soil health of multi-generation apple orchards. Biol. Control.

[b0075] Chase J.M. (2014). Spatial scale resolves the niche versus neutral theory debate. J. Veg. Sci..

[b0080] Chen J., Bittinger K., Charlson E.S., Hoffmann C., Lewis J., Wu G.D., Collman R.G., Bushman F.D., Li H. (2012). Associating microbiome composition with environmental covariates using generalized UniFrac distances. Bioinformatics.

[b0085] Cole J.R., Wang Q., Fish J.A., Chai B., McGarrell D.M., Sun Y., Brown C.T., Porras-Alfaro A., Kuske C.R., Tiedje J.M. (2014). Ribosomal database project: data and tools for high throughput rRNA analysis. Nucleic Acids Res..

[b0090] Deacon L.J., Janie Pryce-Miller E., Frankland J.C., Bainbridge B.W., Moore P.D., Robinson C.H. (2006). Diversity and function of decomposer fungi from a grassland soil. Soil Biol. Biochem..

[b0095] Deakin, G., Tilston, E.L., Bennett, J., Passey, T., Harrison, N., Fernández, F., Xu, X.-M., 2018. Soil microbiome of two apple orchards in the U.K. Data Br. Submitted. 10.1016/j.dib.2018.11.067 PMC6262184 30533450

[b0100] Dequiedt S., Saby N.P.A., Lelievre M., Jolivet C., Thioulouse J., Toutain B., Arrouays D., Bispo A., Lemanceau P., Ranjard L. (2011). Biogeographical patterns of soil molecular microbial biomass as influenced by soil characteristics and management. Glob. Ecol. Biogeogr..

[b0105] Dixon P. (2003). VEGAN, a package of R functions for community ecology. J. Veg. Sci..

[b0110] Docherty K.M., Borton H.M., Espinosa N., Gebhardt M., Gil-Loaiza J., Gutknecht J.L.M., Maes P.W., Mott B.M., Parnell J.J., Purdy G., Rodrigues P.A.P., Stanish L.F., Walser O.N., Gallery R.E. (2015). Key edaphic properties largely explain temporal and geographic variation in soil microbial communities across four biomes. PLoS One.

[b0115] Douhan G.W., Vincenot L., Gryta H.H., Selosse M.-A.M.-A. (2011). Population genetics of ectomycorrhizal fungi: from current knowledge to emerging directions. Fungal Biol..

[b0120] Edgar R.C. (2013). UPARSE: highly accurate OTU sequences from microbial amplicon reads. Nat. Methods.

[b0125] Edgar R.C. (2010). Search and clustering orders of magnitude faster than BLAST. Bioinformatics.

[b0130] Edgar R.C., Flyvbjerg H. (2015). Error filtering, pair assembly and error correction for next-generation sequencing reads. Bioinformatics.

[b0135] Ettema C.H., Wardle D.A. (2002). Spatial soil ecology. Trends Ecol. Evol..

[b0140] FAO, IUSS (2015). World reference base for soil resources 2014: international soil classification system for naming soils and creating legends for soil maps -update 2015.

[b0145] Fierer N. (2017). Embracing the unknown: disentangling the complexities of the soil microbiome. Nat. Rev. Microbiol..

[b0150] Fierer N., Bradford M.A., Jackson R.B. (2007). Toward an ecological classification of soil bacteria. Ecology.

[b0155] Fierer N., Jackson R.B. (2006). The diversity and biogeography of soil bacterial communities. PNAS.

[b0160] Franklin R.B., Mills A.L. (2009). Importance of spatially structured environmental heterogeneity in controlling microbial community composition at small spatial scales in an agricultural field. Soil Biol. Biochem..

[b0165] Franklin R.B., Mills A.L. (2003). Multi-scale variation in spatial heterogeneity for microbial community structure in an eastern Virginia agricultural field. FEMS Microbiol. Ecol..

[b0170] Green J., Bohannan B.J.M. (2006). Spatial scaling of microbial biodiversity. Trends Ecol. Evol..

[b0175] Haichar F. el Z., Marol C., Berge O., Rangel-Castro J.I., Prosser J.I., Balesdent J., Heulin T., Achouak W. (2008). Plant host habitat and root exudates shape soil bacterial community structure. ISME J.

[b0180] Hansen K., Perry B.A., Dranginis A.W., Pfister D.H. (2013). A phylogeny of the highly diverse cup-fungus family Pyronemataceae (Pezizomycetes, Ascomycota) clarifies relationships and evolution of selected life history traits. Mol. Phylogenet. Evol..

[b0185] Harte J., McCarthy S., Taylor K., Kinzig A., Fischer M.L. (1999). Estimating species-area relationships from plot to landscape scale using species spatial-turnover data. Oikos.

[b0190] Hartmann M., Frey B., Mayer J., Mader P., Widmer F. (2015). Distinct soil microbial diversity under long-term organic and conventional farming. ISME J..

[b0195] Hiltner L. (1904). Uber neuere Erfahrungen und Probleme auf dem Gebiete der Bodenbakteriologie unter besonderden berucksichtigung und Brache. Arb. Dtsch. Landwirtsch. Gesellschaft.

[b0200] Janssen P.H. (2006). Identifying the dominant soil bacterial taxa in libraries of 16S rRNA and 16S rRNA genes. Appl. Environ. Microbiol..

[b0205] Kelderer M., Manici L.M., Caputo F., Thalheimer M. (2012). Planting in the “inter-row” to overcome replant disease in apple orchards: a study on the effectiveness of the practice based on microbial indicators. Plant Soil.

[b0210] Koljalg U., Nilsson R.H., Abarenkov K., Tedersoo L., Taylor A.F., Bahram M., Bates S.T., Bruns T.D., Bengtsson-Palme J., Callaghan T.M., Douglas B., Drenkhan T., Eberhardt U., Duenas M., Grebenc T., Griffith G.W., Hartmann M., Kirk P.M., Kohout P., Larsson E., Lindahl B.D., Lucking R., Martin M.P., Matheny P.B., Nguyen N.H., Niskanen T., Oja J., Peay K.G., Peintner U., Peterson M., Poldmaa K., Saag L., Saar I., Schussler A., Scott J.A., Senes C., Smith M.E., Suija A., Taylor D.L., Telleria M.T., Weiss M., Larsson K.H. (2013). Towards a unified paradigm for sequence-based identification of fungi. Mol. Ecol..

[b0215] Kolton M., Sela N., Elad Y., Cytryn E. (2013). Comparative genomic analysis indicates that niche adaptation of terrestrial flavobacteria is strongly linked to plant glycan metabolism. PLoS One.

[b0220] Kuramae E.E., Yergeau E., Wong L.C., Pijl A.S., van Veen J.A., Kowalchuk G.A. (2012). Soil characteristics more strongly influence soil bacterial communities than land-use type. FEMS Microbiol. Ecol..

[b0225] Lauber C.L., Strickland M.S., Bradford M.A., Fierer N. (2008). The influence of soil properties on the structure of bacterial and fungal communities across land-use types. Soil Biol. Biochem..

[b0230] Lemanceau P., Maron P.-A., Mazurier S., Mougel C., Pivato B., Plassart P., Ranjard L., Revellin C., Tardy V., Wipf D. (2015). Understanding and managing soil biodiversity: a major challenge in agroecology. Agron. Sustain. Dev..

[b0235] Love M.I., Huber W., Anders S. (2014). Moderated estimation of fold change and dispersion for RNA-seq data with DESeq2. Genome Biol..

[b0240] Lundell T.K., Mäkelä M.R., de Vries R.P., Hildén K.S., Martin F.M. (2014). Genomics, lifestyles and future prospects of wood-decay and litter-decomposing Basidiomycota. Advances in Botanical Research.

[b0245] MAFF, ADAS (1986). The Analysis of Agricultural Materials: Reference Book 427.

[b0250] Martiny J.B.H., Eisen J.A., Penn K., Allison S.D., Horner-Devine M.C. (2011). Drivers of bacterial beta-diversity depend on spatial scale. Proc. Natl. Acad. Sci. U. S. A..

[b0255] Mazzola M., Hewavitharana S.S., Strauss S.L. (2015). Brassica seed meal soil amendments transform the rhizosphere microbiome and improve apple production through resistance to pathogen reinfestation. Phytopathology.

[b0260] McMurdie P.J., Holmes S. (2013). Phyloseq: An R package for reproducible interactive analysis and graphics of microbiome census data. PLoS One.

[b0265] McMurdie P.J., Holmes S. (2014). Waste not, want not: why rarefying microbiome data is inadmissible. PLoS Comput. Biol..

[b0270] Miller D.D., Domoto P.A., Walker C. (1985). Mycorrhizal fungi at eighteen apple rootstock plantings in the United States. New Phytol..

[b0275] Morlon H., Chuyong G., Condit R., Hubbell S., Kenfack D., Thomas D., Valencia R., Green J.L. (2008). A general framework for the distance-decay of similarity in ecological communities. Ecol. Lett..

[b0280] Mouhamadou B., Molitor C., Baptist F., Sage L., Clément J.-C., Lavorel S., Monier A., Geremia R.A. (2011). Differences in fungal communities associated to *Festuca paniculata* roots in subalpine grasslands. Fungal Divers..

[b0285] Nakagawa T., Nagaoka T., Taniguchi S., Miyaji T., Tomizuka N. (2004). Isolation and characterization of psychrophilic yeasts producing cold-adapted pectinolytic enzymes. Lett. Appl. Microbiol..

[b0290] Naveed M., Herath L., Moldrup P., Arthur E., Nicolaisen M., Norgaard T., Ferré T.P.A., de Jonge L.W. (2016). Spatial variability of microbial richness and diversity and relationships with soil organic carbon, texture and structure across an agricultural field. Appl. Soil Ecol..

[b0295] Nekola J.C., White P.S. (1999). The distance decay of similarity in biogeography and ecology. J. Biogeogr..

[b0300] Nilsson R.H., Veldre V., Wang Z., Eckart M., Branco S., Hartmann M., Quince C., Godhe A., Bertrand Y., Alfredsson J.F., Larsson K.-H., Kõljalg U., Abarenkov K. (2011). A note on the incidence of reverse complementary fungal ITS sequences in the public sequence databases and a software tool for their detection and reorientation. Mycoscience.

[b0305] Paradis E., Claude J., Strimmer K. (2004). APE: analyses of phylogenetics and evolution in R language. Bioinformatics.

[b0310] Peruzzi E., Franke-Whittle I.H., Kelderer M., Ciavatta C., Insam H. (2017). Microbial indication of soil health in apple orchards affected by replant disease. Appl. Soil Ecol..

[b0315] Qin S., Jiao K., He J., Lyu D. (2016). Forage crops alter soil bacterial and fungal communities in an apple orchard. Acta Agric. Scand. Sect. B-Soil Plant Sci..

[b0320] R Core Development Team. R: A language and environment for statistical computing, 2008.

[b0325] Rich J.J., Heichen R.S., Bottomley P.J., Cromack K., Myrold D.D. (2003). Community composition and functioning of denitrifying bacteria from adjacent meadow and forest soils. Appl. Environ. Microbiol..

[b0330] Rousk J., Baath E., Brookes P.C., Lauber C.L., Lozupone C., Caporaso J.G., Knight R., Fierer N. (2010). Soil bacterial and fungal communities across a pH gradient in an arable soil. ISME J..

[b0335] Sati S.C., Arya P. (2010). Assessment of root endophytic aquatic hyphomycetous fungi on plant growth. Symbiosis.

[b0340] Simon S., Lesueur-Jannoyer M., Plénet D., Lauri P.-É., Le Bellec F. (2017). Methodology to design agroecological orchards: Learnings from on-station and on-farm experiences. Eur. J. Agron..

[b0345] Singh B.K., Nunan N., Ridgway K.P., McNicol J., Young J.P.W., Daniell T.J., Prosser J.I., Millard P. (2008). Relationship between assemblages of mycorrhizal fungi and bacteria on grass roots. Environ. Microbiol..

[b0350] St. Laurent A., Merwin I.A., Thies J.E. (2008). Long-term orchard groundcover management systems affect soil microbial communities and apple replant disease severity. Plant Soil.

[b0355] St. Laurent A., Merwin I., Fazio G., Thies J., Brown M. (2010). Rootstock genotype succession influences apple replant disease and root-zone microbial community composition in an orchard soil. Plant Soil.

[b0360] Stursová M., Zifčáková L., Leigh M., Burgess R., Baldrian P. (2012). Cellulose utilization in forest litter and soil: identification of bacterial and fungal decomposers. FEMS Microbiol. Ecol..

[b0365] Tewoldemedhin Y.T., Mazzola M., Labuschagne I., McLeod A. (2011). A multi-phasic approach reveals that apple replant disease is caused by multiple biological agents, with some agents acting synergistically. Soil Biol. Biochem..

[b0370] Tilston E.L., Halpin C., Hopkins D.W. (2014). Simultaneous down-regulation of enzymes in the phenylpropanoid pathway of plants has aggregated effects on rhizosphere microbial communities. Biol. Fertil. Soils.

[b0375] Vellend M. (2010). Conceptual synthesis in community ecology. Q. Rev. Biol..

[b0380] Wei F., Fan R., Passey T., Hu X.-P., Xu X. (2016). Identification of candidate soil microbes responsible for small-scale heterogeneity in strawberry plant vigour. J. Integr. Agric..

[b0385] Wheeler, R.E., Permutation tests for linear models in R, https://cran.r-project.org/web/packages/lmPerm/vignettes/lmPerm.pdf, 2016.

[b0390] Wheeler T.J., Eddy S.R. (2013). nhmmer: DNA homology search with profile HMMs. Bioinformatics.

[b0395] Wong M.K.M., Hyde K.D. (2001). Diversity of fungi on six species of Gramineae and one species of Cyperaceae in Hong Kong. Mycol. Res..

[b0400] Yao S., Merwin I.A., Abawi G.S., Thies J.E. (2006). Soil fumigation and compost amendment alter soil microbial community composition but do not improve tree growth or yield in an apple replant site. Soil Biol. Biochem..

[b0405] Zhou J., Xia B., Huang H., Palumbo A.V., Tiedje J.M. (2004). Microbial diversity and heterogeneity in sandy subsurface soils. Appl. Environ. Microbiol..

