# Supplementary material for: Spatial structuring of soil microbial communities in commercial apple orchards
Source: Appl Soil Ecol. 2018 Sep;130:1–12. doi: 10.1016/j.apsoil.2018.05.015 (PMC6102658; doi:10.1016/j.apsoil.2018.05.015)
Supplement: Supplementary data 1 — Supplementary material. [file mmc1.docx]

# Supplementary Figures


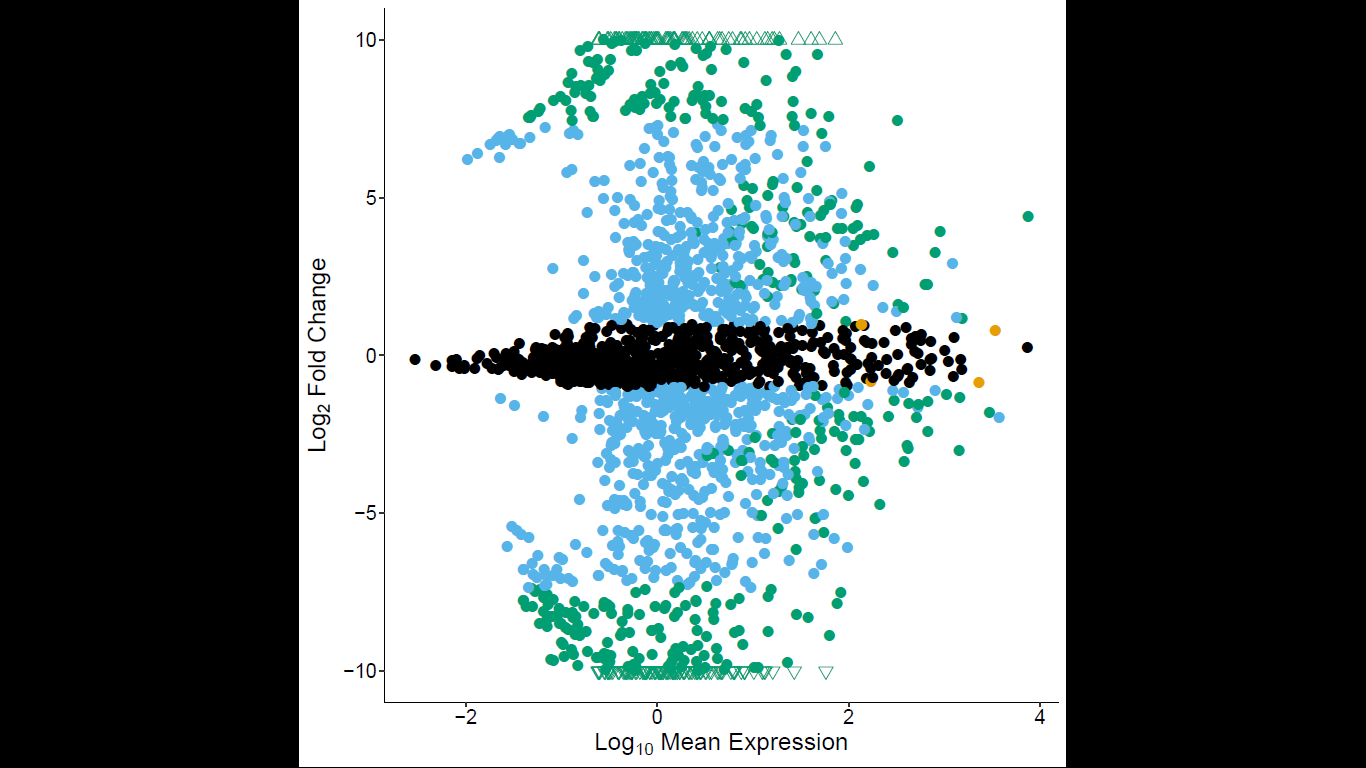

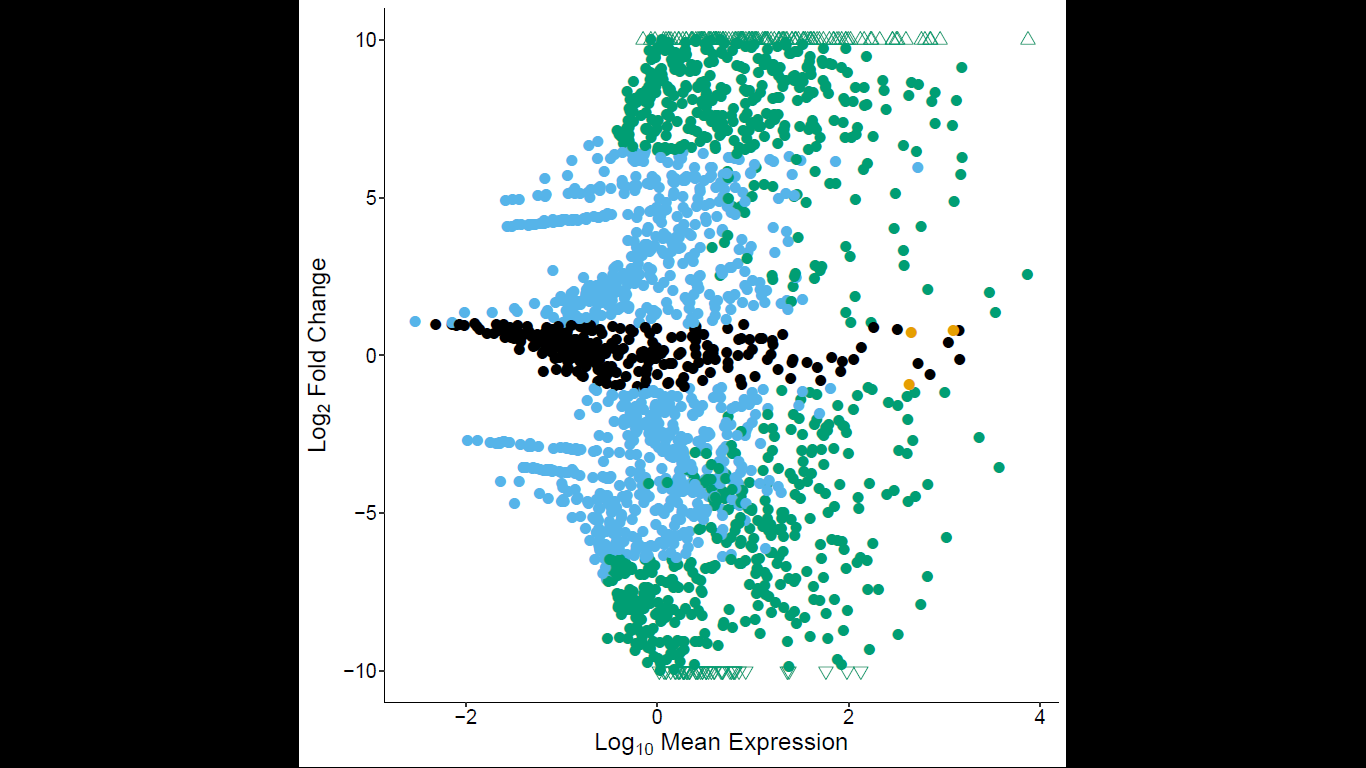

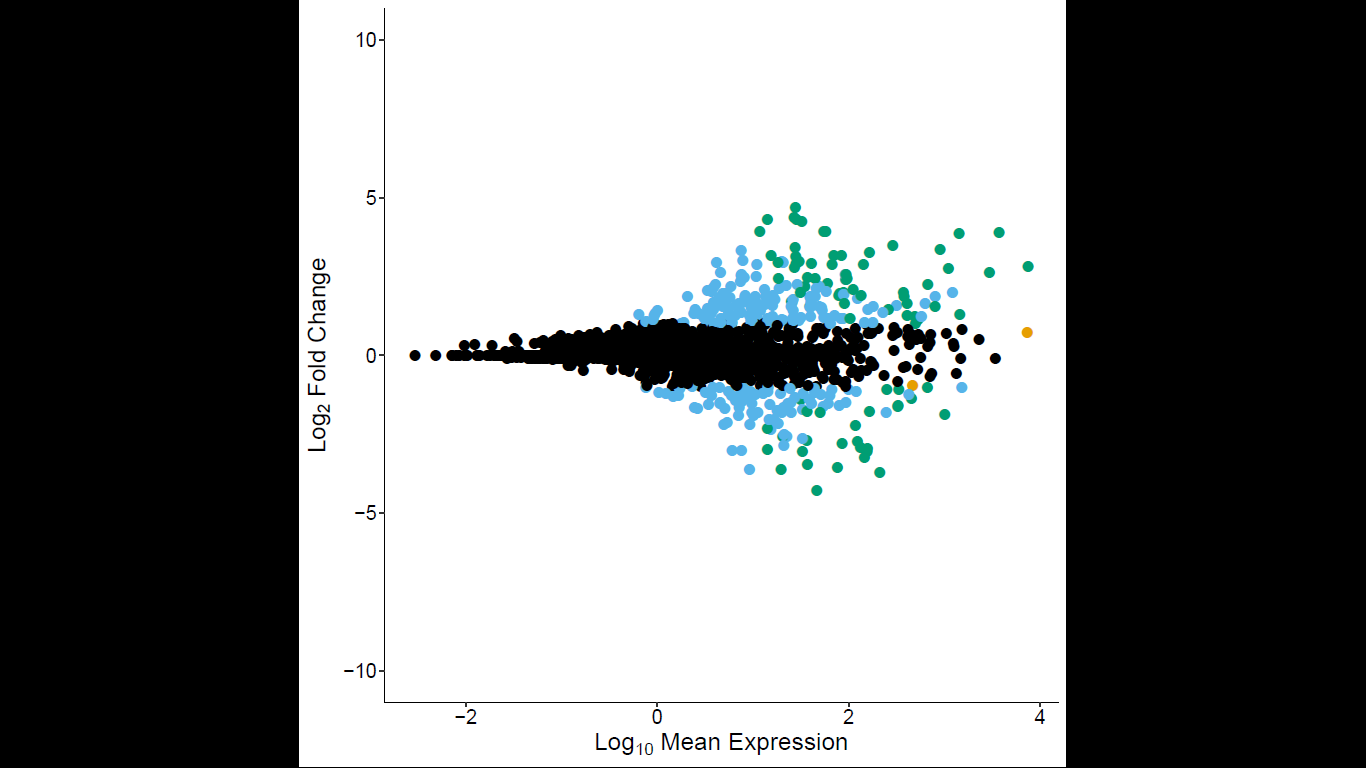


Log_10_ mean abundance

Log_10_ mean abundance

Log_10_ mean abundance

Not sig.

FC > 2

*p* <= 0.05

*p* <= 0.05

and FC > 2

F

E

D

C

B

A


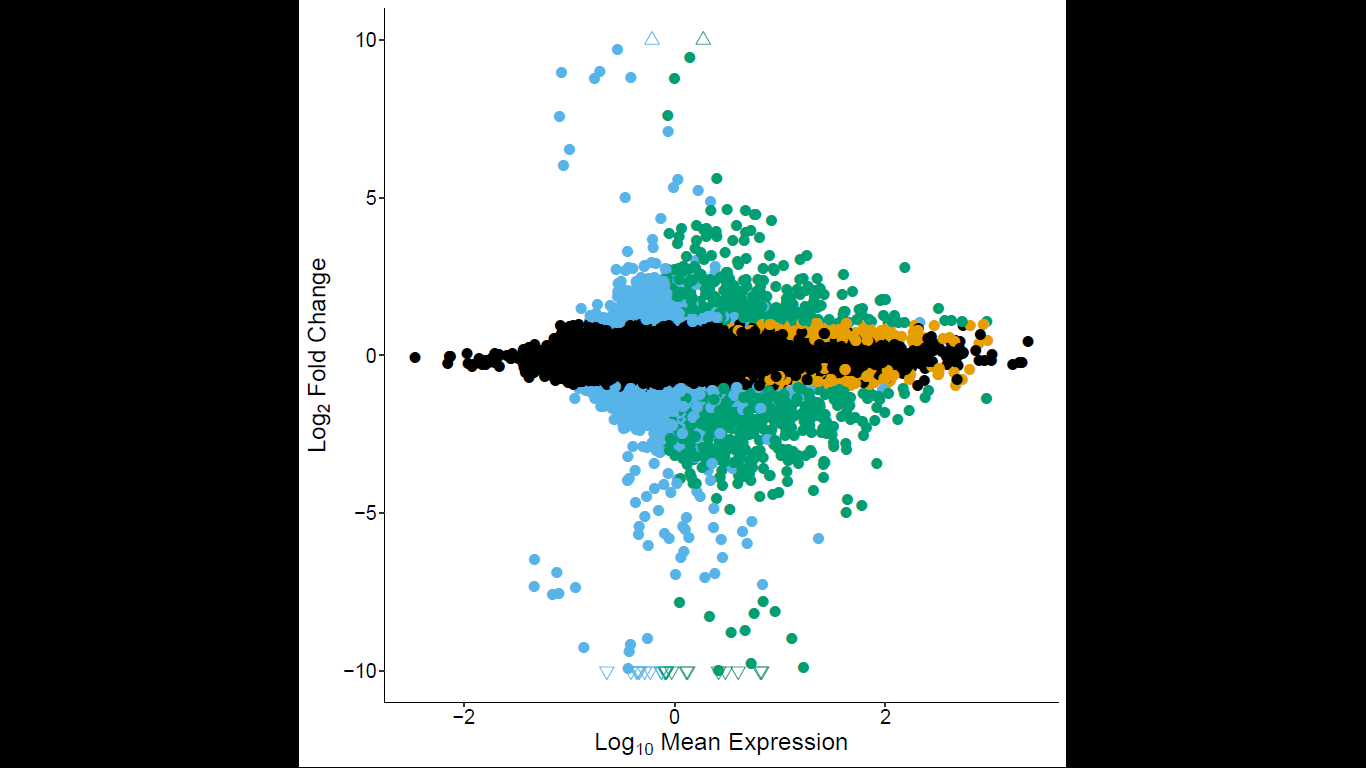

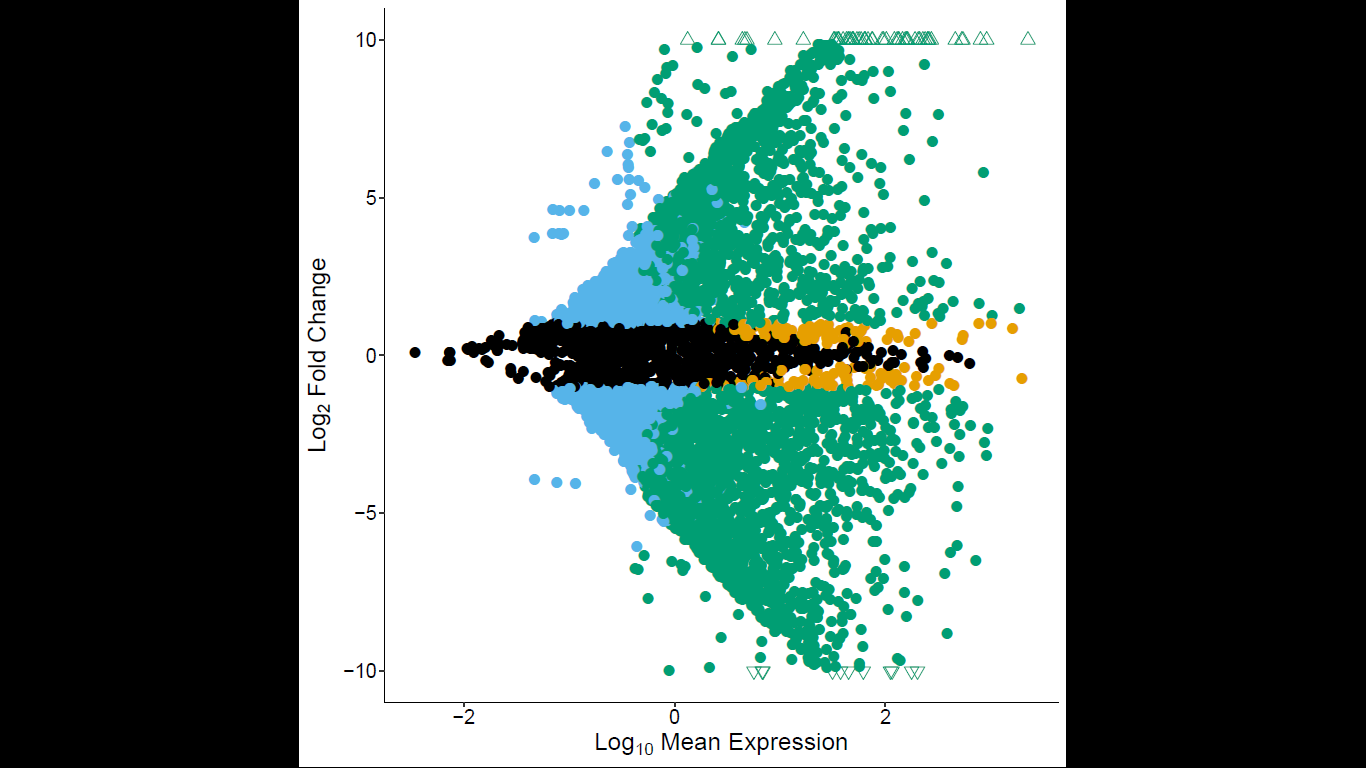

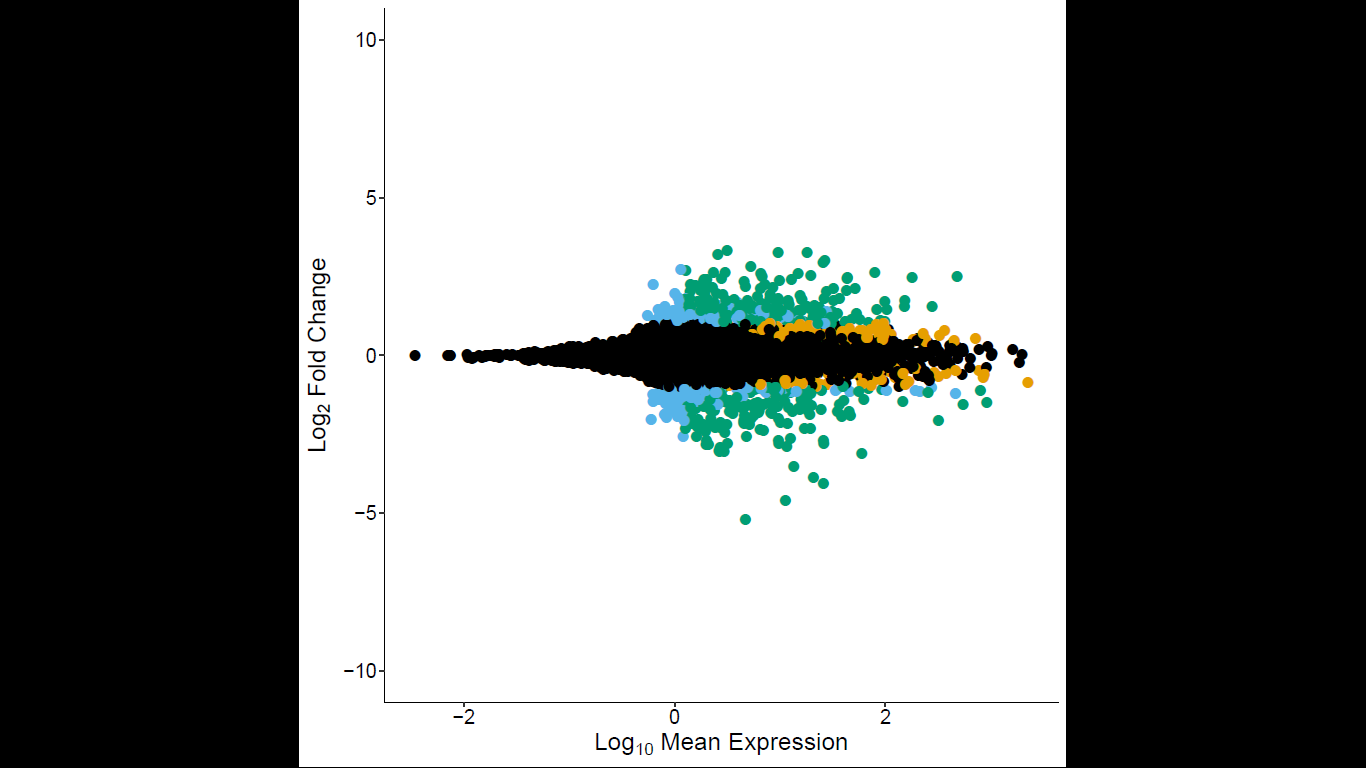


Log_10_ mean abundance

Log_10_ mean abundance

Log_10_ mean abundance

**Figure S1.** Fungal and bacterial MA plots where the Log_2_ fold change of relative fungal abundance was plotted against average (Log_10_) relative abundance for each OTU: between vegetation type (tree station vs grass aisle, **A**), between orchards (**B**), and for orchard x vegetation type interaction (**C**). Corresponding Figs for bacteria are shown in (**D**), (**E**) and (**F**)


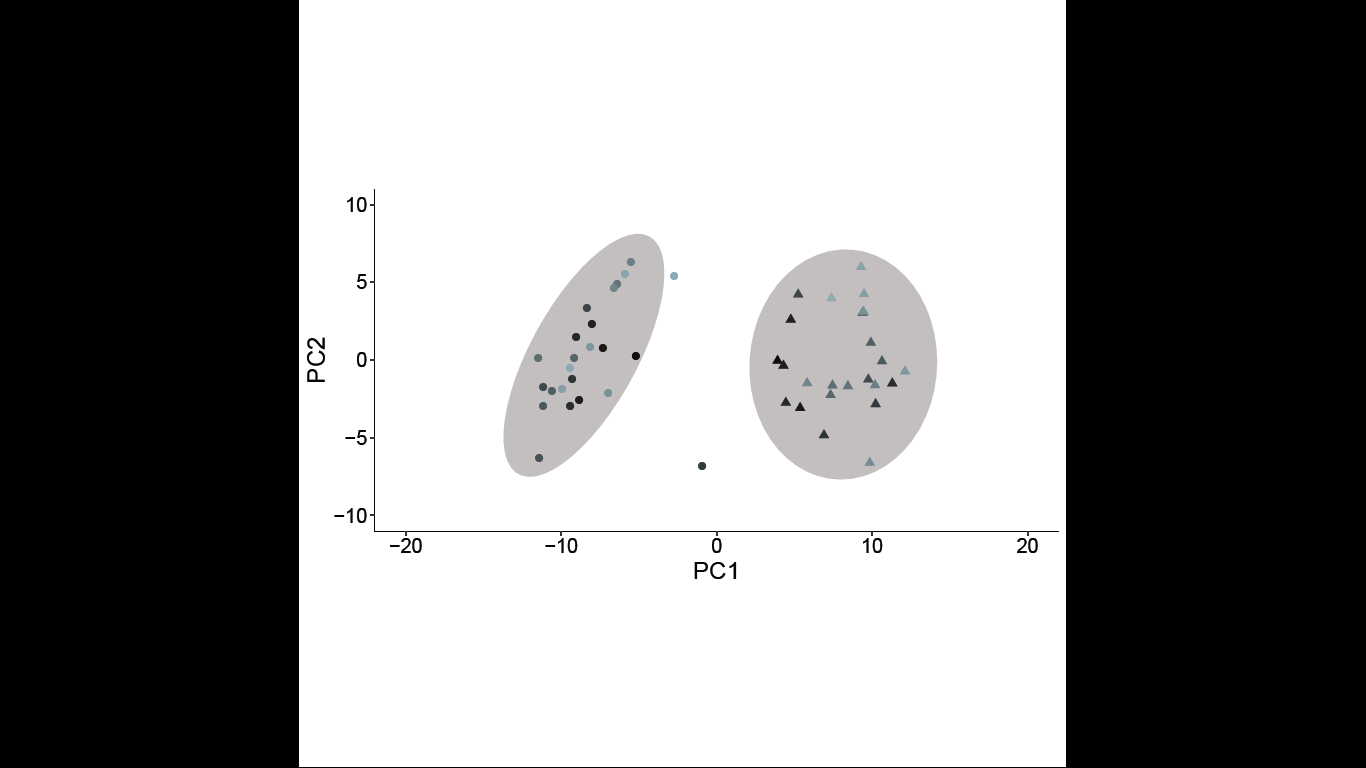

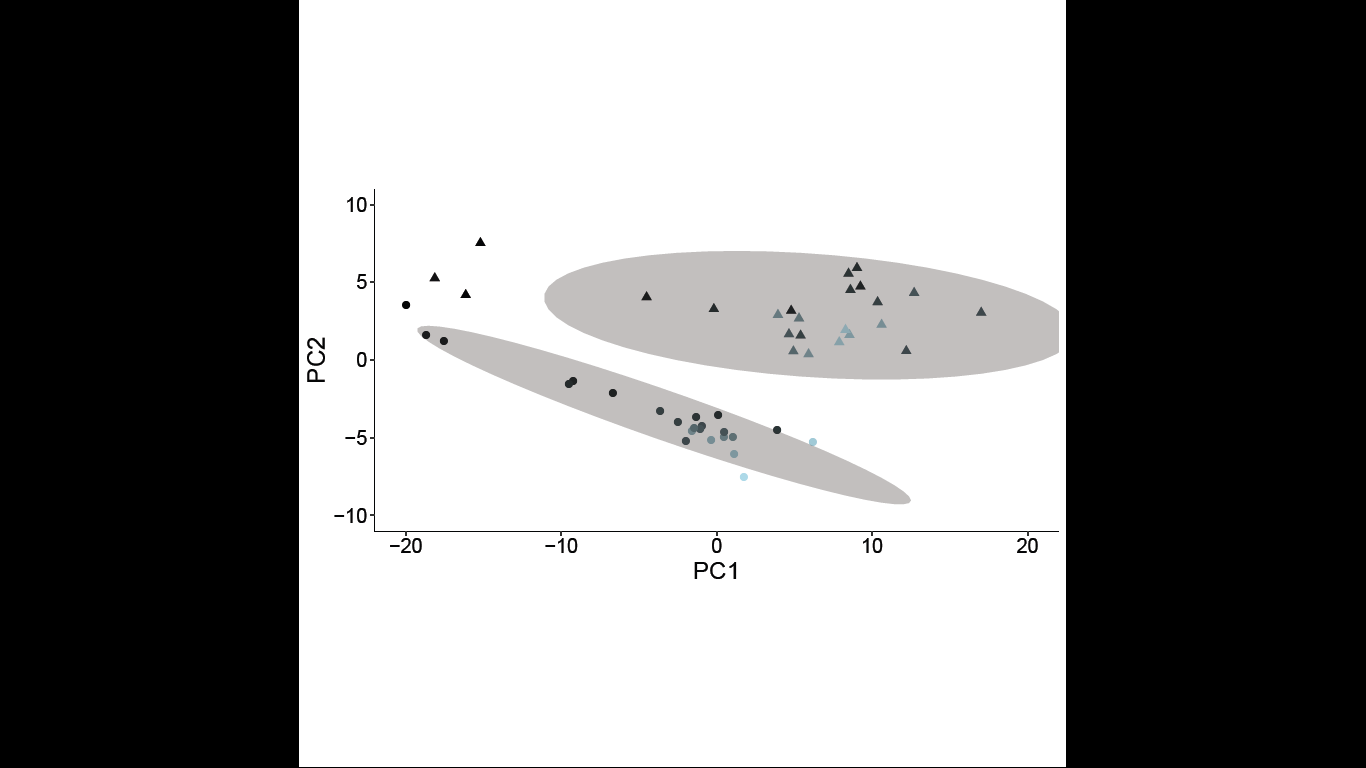


A

C


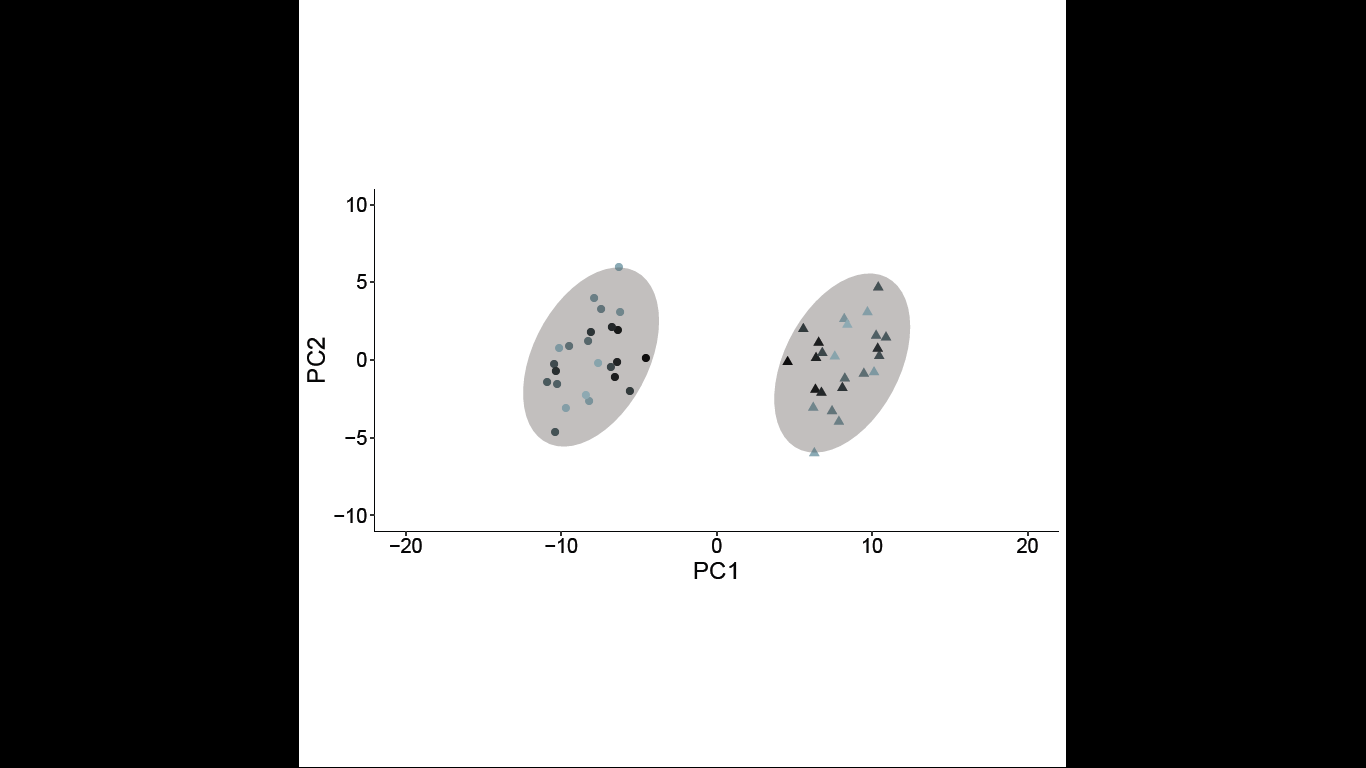

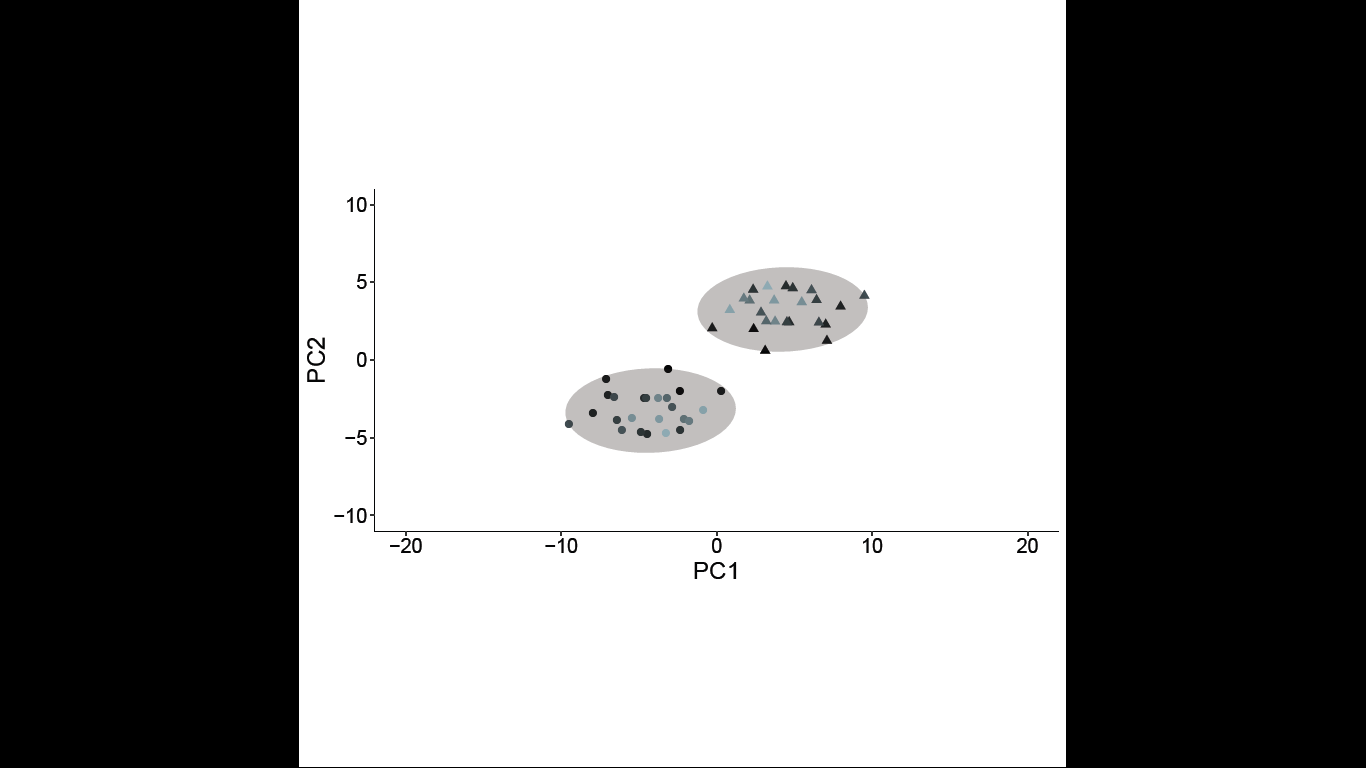

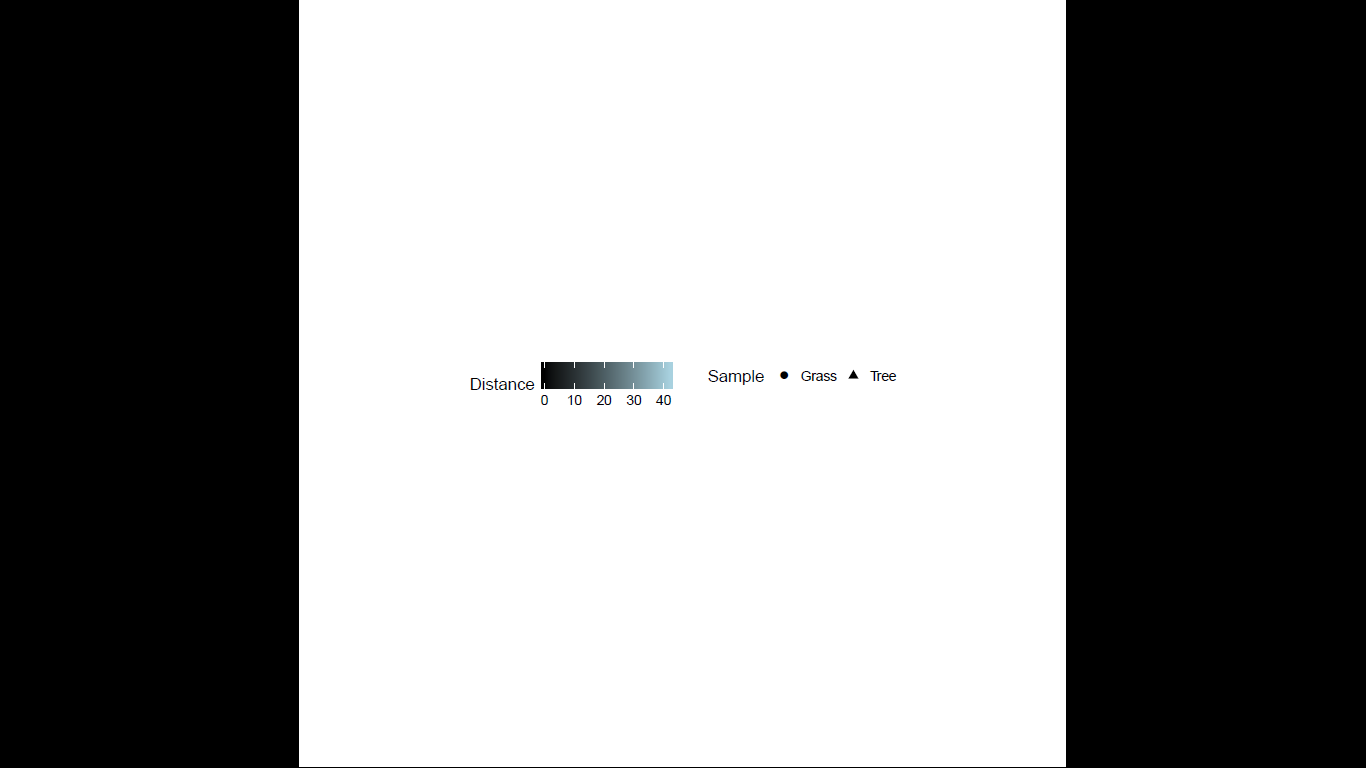


B

D

**Figure S2.** Principal component analysis of bacterial OTUs, conducted for each orchard separately. All graphs show PC1 vs PC2. Point shape represents tree station (triangle) or grass aisle (circle) samples and point colour represents the actual physical location of each sample along the sampling row direction within an orchard. Clusters with >9 5% confidence have been coloured in grey. (**A)** PC scores of variance stabilised OTUs counts for the *dessert* orchard, (**B**) PC scores after removal of the spatial location effect for the *dessert* orchard, (**C**) PC scores of variance stabilised OTUs counts for the *cider* orchard, and (**D**) PC scores after removal of the spatial location effect for the *cider* orchard


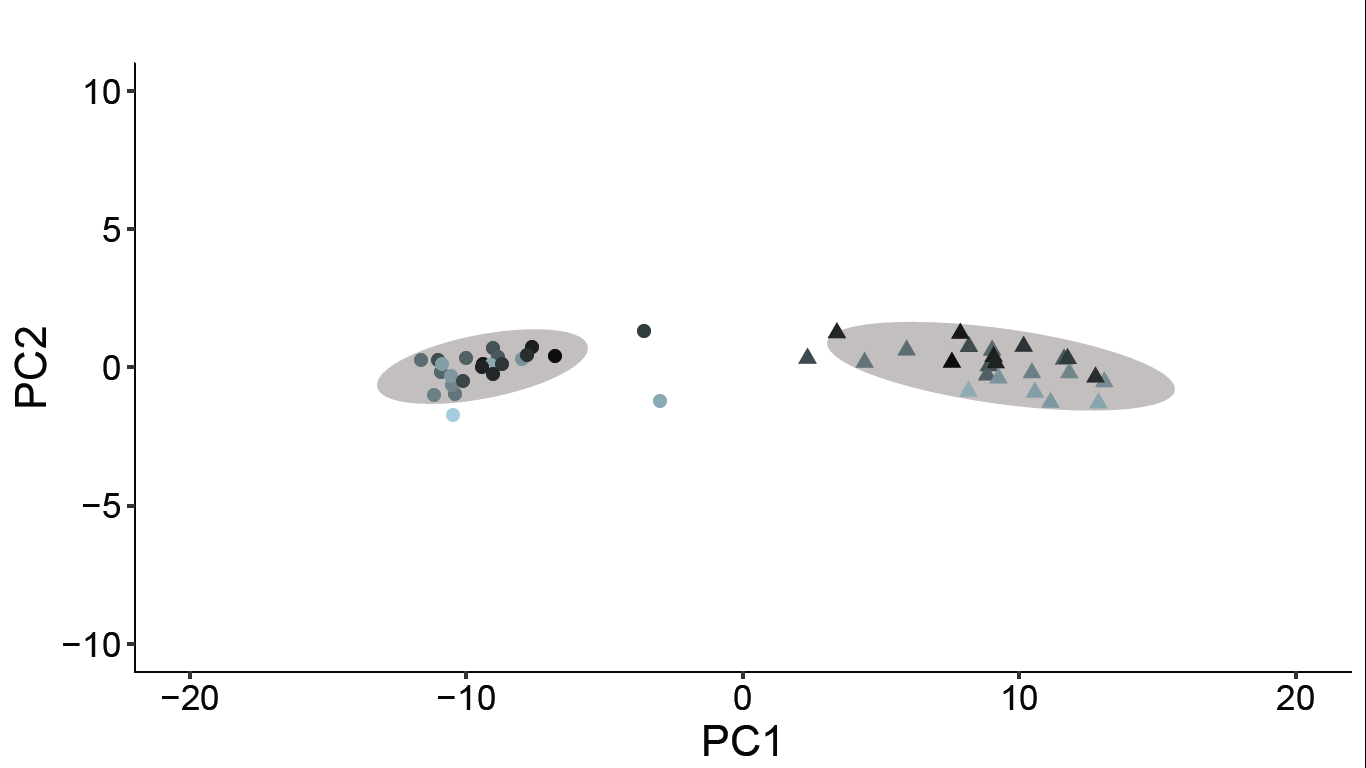

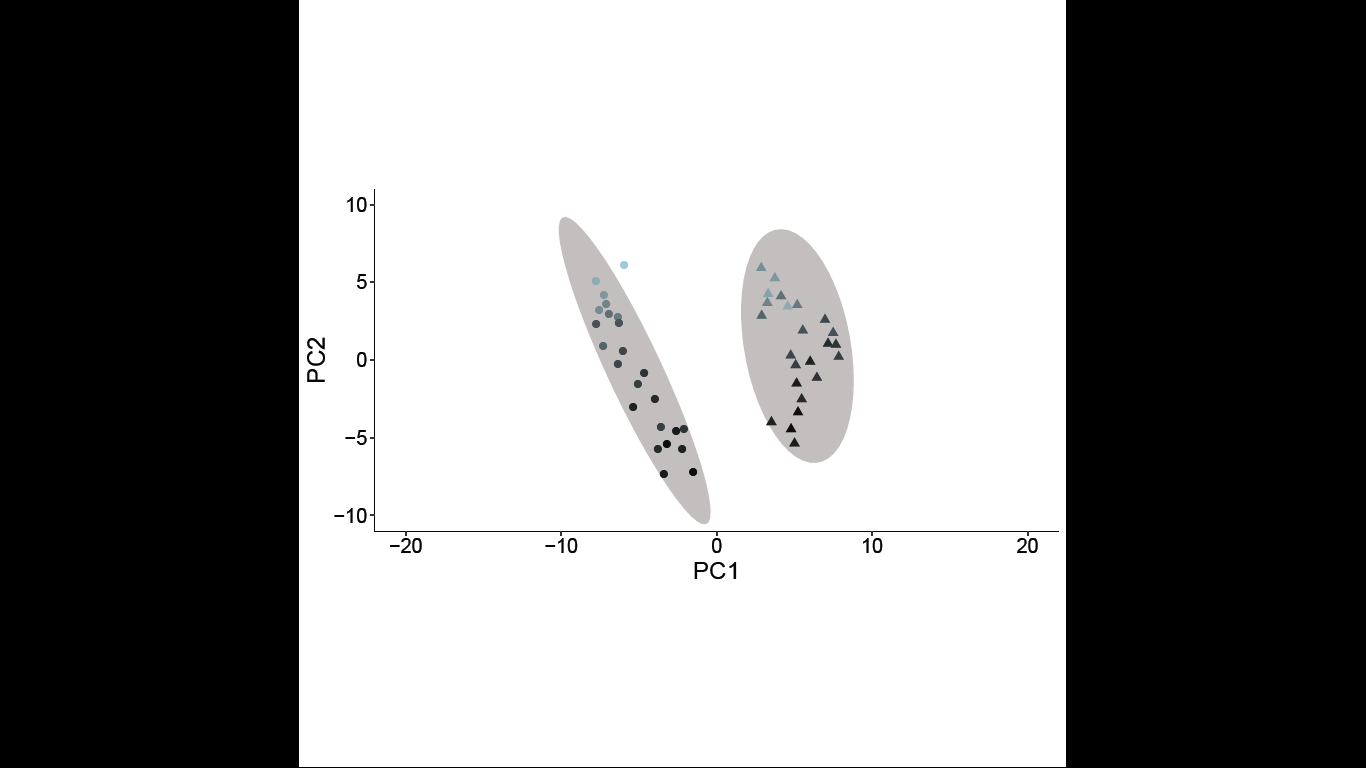


A

BB

CC


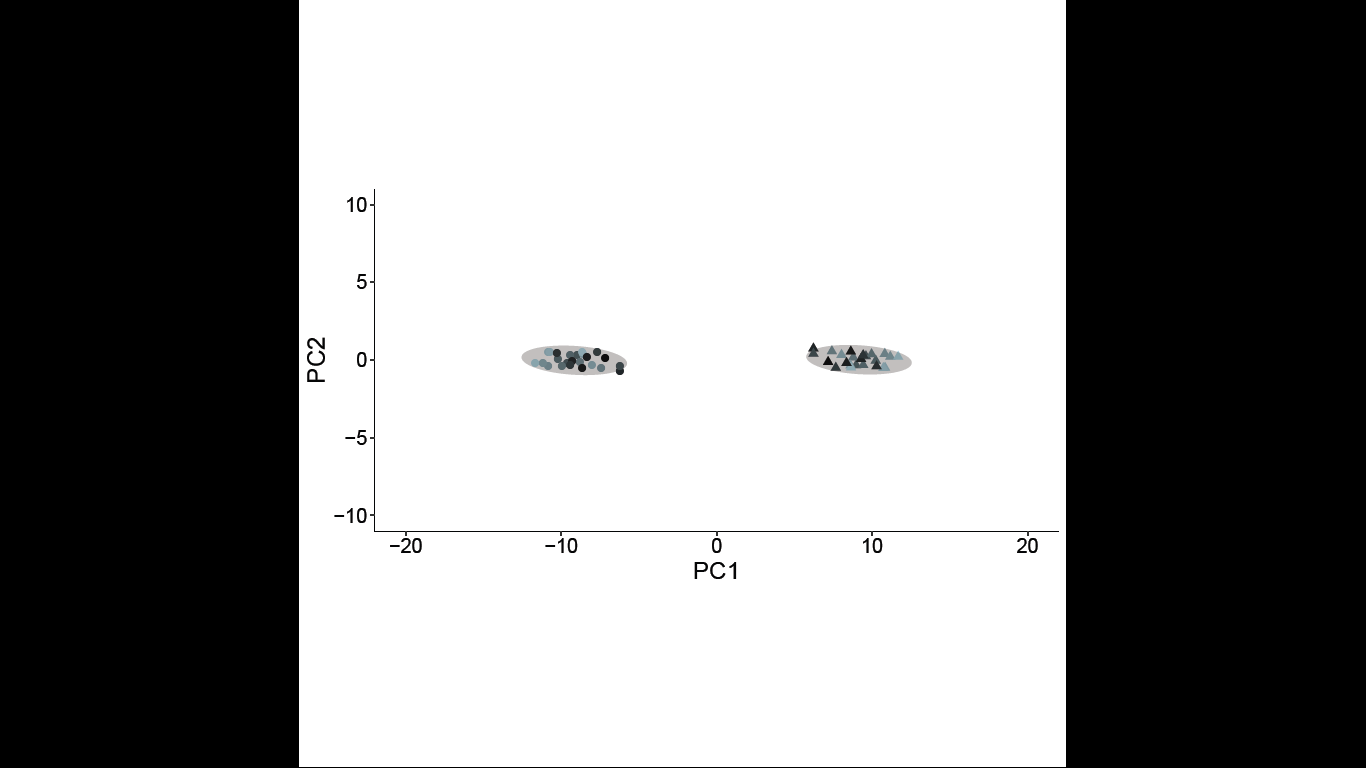

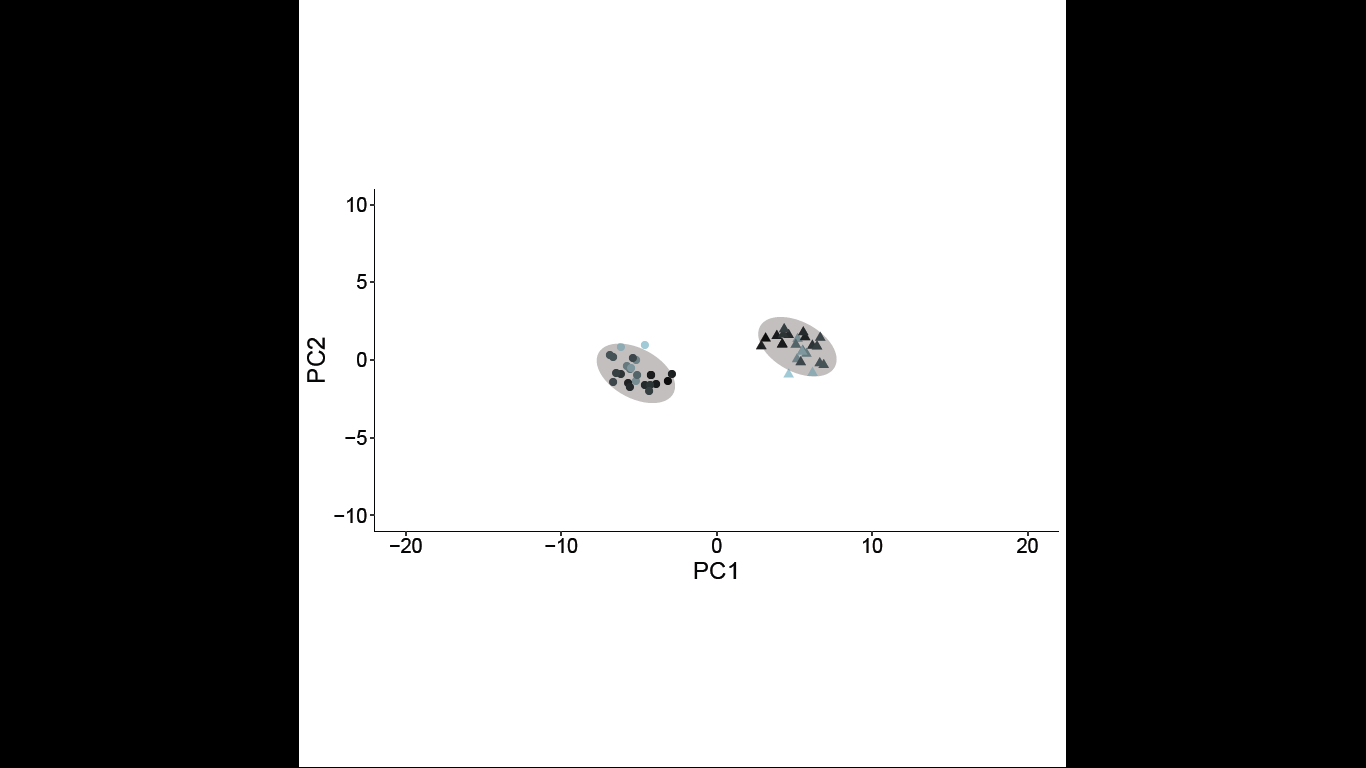


D


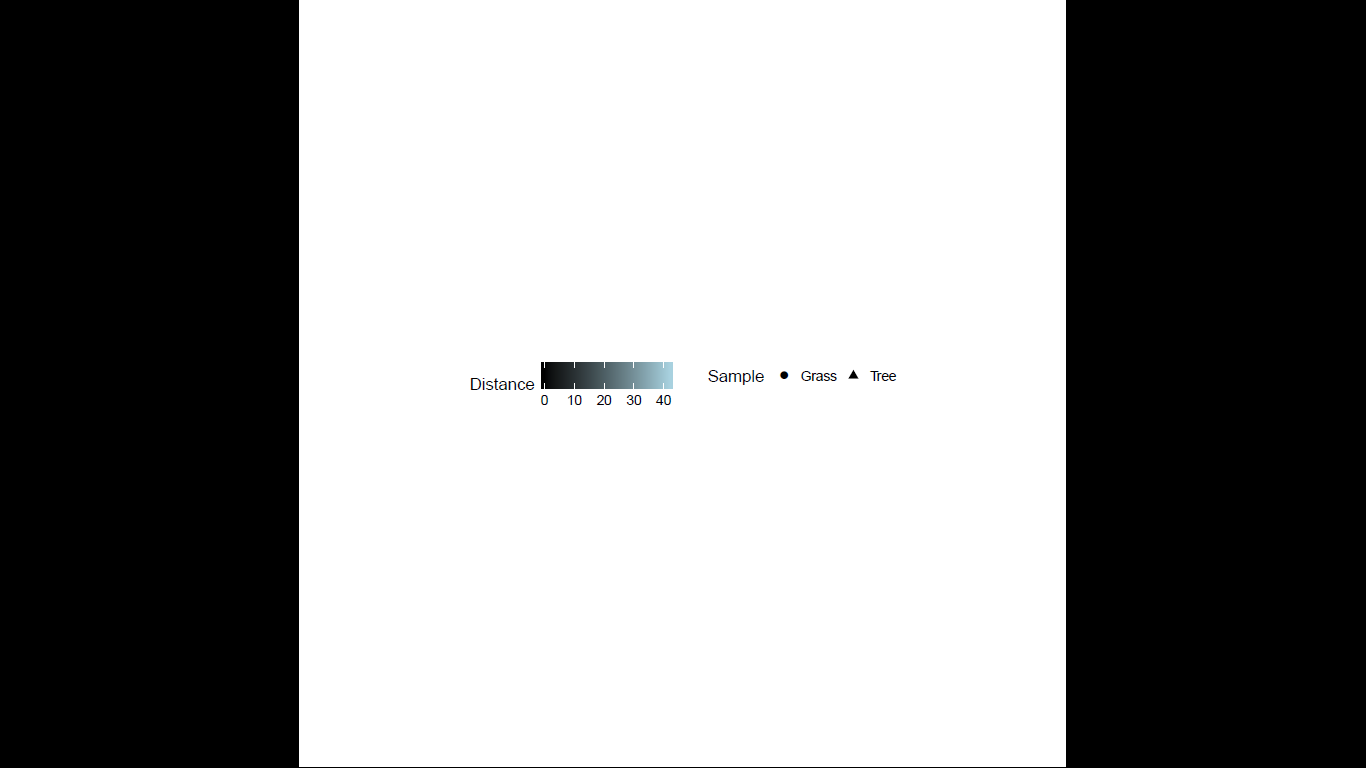


**Figure S3.** Principal component analysis of fungal OTUs, conducted for each orchard separately. All graphs show PC1 vs PC2. Point shape represents tree station (triangle) or grass aisle (circle) samples and point colour represents the actual physical location of each sample along the sampling row direction within an orchard. Clusters with >95% confidence have been coloured in grey. (**A)** PC scores of variance stabilised OTUs counts for the *dessert* orchard, (**B**) PC scores after removal of the spatial location effect for the *dessert* orchard, (**C**) PC scores of variance stabilised OTUs counts for the *cider* orchard, and (**D**) PC scores after removal of the spatial location effect for the *cider* orchard
