# Supplementary material for: Spatial structuring of soil microbial communities in commercial apple orchards
Source: Appl Soil Ecol. 2018 Sep;130:1–12. doi: 10.1016/j.apsoil.2018.05.015 (PMC6102658; doi:10.1016/j.apsoil.2018.05.015)
Supplement: Supplementary data 2 — Data profile. [file mmc2.docx]

**Title**: Soil microbiome of two apple orchards in the U.K.

**Authors**: Greg Deakin, Emma L. Tilston, Julie Bennett, Tom Passey, Nicola Harrison, Felicidad Fernández-Fernández, Xiangming Xu

**Affiliations**: NIAB EMR, East Malling, West Malling, Kent ME19 6BJ, UK

**Contact email**: greg.deakin@emr.ac.uk

# Abstract

The microbial communities in two apple orchards were characterised using amplicon-based metabarcoding. Samples were taken from tree station locations along a linear transect and from adjacent grass aisles, at both orchards. Comparison was made between the communities occurring at tree station locations and the grass aisles, and between orchards. Further discussion of these datasets is given in Deakin, Tilston *et al*. (2018) [1].

**Specifications Table**

| Subject area | Biology |
| --- | --- |
| More specific subject area | Soil microbial ecology, metabarcoding, spatial correlation |
| Type of data | Text files (DNA sequence and tab separated abundance data) and tables |
| How data was acquired | Illumina MiSeq with v3 chemistry |
| Data format | Raw data and analysed data |
| Experimental factors | Fungal and bacterial soil communities from two apple orchards from both managed and unmanaged soil |
| Experimental features | Operation taxonomic units quantitated using metabarcoding of ITS (fungal) and 16S (bacterial) ribosomal regions. Three samples measured for each sample point and mean used for quantification. |
| Data source location | Dessert Orchard: UK lat. 51.210596, long. 0.601664  Cider orchard: UK lat. 52.251020, long. -2.301711 |
| Data accessibility | The data are available with this article |

## ****Value of the data****

These metabarcoding data include both fungal and bacterial communities from two long standing apple orchards in the U.K. As such they offer a wealth of future opportunities to:

1. aid in identifying common microbial communities between apple orchards
2. make comparison of microbial communities under different soil management practices, e.g. long term perennial crops vs annual crops
3. help identify best management practices for enhancing soil microbial communities

# Data

The datasets provided with this article give abundance and comparative analyses of the bacterial and fungal communities found in a U.K. dessert apple and a cider apple orchard. As a multi-layer assemblage of plants in a long-term complex spatial arrangement, commercial apple orchards are an interesting agroecosystem in which to study plant-soil-microbial interactions. The data contain both fungal and bacterial operational taxonomic units (OTUs) found at tree stations and adjacent (approx. 2 m) grass aisle between tree rows. Further context to these data (e.g. soil description and management practice) are given in [1]. Supplementary Files 1, 2 and 3 contain OTU sequence information, OTU taxonomy and raw sample abundance for the OTUs respectively for fungal communities. Supplementary files 4, 5 and 6 present the same for bacterial communities. The column headers in supp. files 3 and 6 provide sample metadata (C/D cider or dessert, Y/N tree station or grass aisle, 1-24 sample location, a/b/c sample replicate). Table 1 provides a summary of the sequencing data and Table 2 a summary of the OTU taxonomic data. The top 20 by abundance fungal and bacterial OTUs, which differed significantly between tree station and grass aisle locations, are listed in Tables 3 and 4 respectively. The numbers of OTUs, aggregated at both the phylum and class levels, which differed between tree station and grass aisle, between orchards or between tree station and grass aisle at each orchard, are given in Tables 5 (fungal phylum rank), 6 (bacterial phylum), 7 (fungal class) and 8 (bacterial class).

| **Table 1.** The number of raw reads, reads aligned to OTUs and OTUs from each kingdom, summed for all samples and for each orchard. | | | | | |
| --- | --- | --- | --- | --- | --- |
|  |  | Total reads per sample | Reads aligned to OTUs | Total OTUs | OTUs > 5 reads |
| All | Fungi | 12456087 | 7016415 (56.3%) | 2132 | 2067 |
|  | Bacteria | 13562736 | 9734624 (71.8%) | 6392 | 6167 |
| Cider | Fungi | 5357051 | 3069449 (57.3%) | 1552 | 1394 |
|  | Bacteria | 7000356 | 5069312 (72.4%) | 5786 | 4752 |
| Dessert | Fungi | 7099036 | 3946966 (55.6%) | 1638 | 1371 |
|  | Bacteria | 6562380 | 4665312 (71.1%) | 4984 | 4472 |

| **Table 2.** The percentage of OTUs which could be classified at the given taxonomic rank by the UTAX algorithm at the confidence level of 0.65. | | | | | | | |
| --- | --- | --- | --- | --- | --- | --- | --- |
|  | Kingdom | Phylum | Class | Order | Family | Genus | Species |
| Fungi | 100 | 63.7 | 44.7 | 36.8 | 28.3 | 17.2 | 6.1 |
| Bacteria | 95.8 | 76.7 | 51.1 | 21.4 | 12.7 | 14.7 | NA |

| **Table 3.** The top 20 (by abundance) fungal OTUs with higher abundance in grass aisles (positive fold change) or tree stations (negative fold change) and with absolute fold change > 2 and Benjamini-Hochberg corrected *p* ≤ 0.05. | | | |
| --- | --- | --- | --- |
| Species/taxa† | Base mean | Fold change | P value |
| Eurotiomycetes(c) | 7488.35 | 20.87 | 1.55x10^-6^ |
| Eurotiomycetes(c) | 903.59 | 15.27 | 1.85x10^-4^ |
| Eurotiomycetes(c) | 797.77 | 9.48 | 4.16x10^-3^ |
| Mortierellaceae(f) | 664.58 | 4.74 | 4.88x10^-7^ |
| Fungi(k) | 627.46 | 4.69 | 2.42x10^-2^ |
| Monodictys(g) | 328.51 | 3.07 | 1.44x10^-2^ |
| Fungi(k) | 371.91 | 2.88 | 1.06x10^-2^ |
| *Mortierella exigua* | 1523.28 | 2.25 | 1.30x10^-2^ |
| *Cryptococcus aerius* | 1048.08 | -2.37 | 4.39x10^-3^ |
| *Ilyonectria macrodidyma* | 1439.95 | -2.51 | 1.30x10^-4^ |
| Tetracladium(g) | 668.40 | -2.74 | 2.03x10^-3^ |
| Trichoderma(g) | 423.53 | -2.88 | 2.01x10^-2^ |
| Ascomycota(p) | 536.66 | -2.93 | 7.11x10^-3^ |
| Ascomycota(p) | 2940.50 | -3.48 | 1.05x10^-2^ |
| Ascomycota(p) | 510.30 | -3.91 | 8.78x10^-4^ |
| Pyronemataceae(f) | 672.12 | -5.3 | 3.52x10^-4^ |
| Pichia(g) | 406.19 | -7.23 | 1.61x10^-11^ |
| *Mrakia frigida* | 417.63 | -7.74 | 3.40x10^-8^ |
| Pyronemataceae(f) | 1418.61 | -8.03 | 2.93x10^-10^ |
| Dothideomycetes(o) | 378.39 | -10.38 | 3.40x10^-10^ |
| †The lowest assignable taxonomic rank with a UTAX confidence ≥ 0.65. | | | |

| **Table 4.** The top 20 (by abundance) bacterial OTUs with higher abundance in grass aisles (positive fold change) or tree stations (negative fold change) and with absolute fold change > 2 and Benjamini-Hochberg corrected *p* ≤ 0.05. | | | |
| --- | --- | --- | --- |
| Genus/taxa† | Base mean | Fold Change | P value |
| Deltaproteobacteria(c) | 151.68 | 6.90 | 3.64x10^-29^ |
| Acidobacteria Group3(c) | 315.87 | 2.76 | 9.69x10^-8^ |
| Myxococcales(o) | 121.44 | 2.39 | 1.06x10^-13^ |
| Gammaproteobacteria(c) | 421.41 | 2.16 | 4.06x10^-10^ |
| Bacteroidetes incertae sedis(c) | 364.51 | 2.15 | 1.64x10^-6^ |
| Acidobacteria Group5(c) | 110.04 | 2.12 | 3.97x10^-7^ |
| Acidobacteria Group1(c) | 910.60 | 2.11 | 2.79x10^-2^ |
| Terrimonas | 529.47 | 2.11 | 2.00x10^-3^ |
| Rhizobiales(c) | 113.99 | 2.10 | 3.77x10^-9^ |
| Betaproteobacteria | 132.13 | 2.06 | 4.40x10^-8^ |
| Acidobacteria Group6(c) | 105.82 | 2.05 | 8.31x10^-4^ |
| Acidobacteria Group6 | 150.35 | 2.03 | 1.58x10^-5^ |
| Xanthobacteraceae(f) | 146.35 | -2.06 | 3.95x10^-7^ |
| Flavobacterium | 254.87 | -2.16 | 4.59x10^-4^ |
| Skermanella | 151.03 | -2.30 | 6.61x10^-6^ |
| Gemmatimonadetes(p) | 106.09 | -2.30 | 6.74x10^-9^ |
| Novosphingobium | 236.82 | -2.53 | 2.20x10^-8^ |
| Pseudomonas | 899.89 | -2.57 | 1.44x10^-8^ |
| Flavobacterium | 166.80 | -3.35 | 3.83x10^-7^ |
| Flavobacterium | 130.38 | -4.13 | 1.50x10^-10^ |
| †The lowest assignable taxonomic rank with a UTAX confidence ≥ 0.65. | | | |

| **Table 5.** The number of fungal OTUs with differential abundance (Benjamini-Hochberg corrected *p* ≤ 0.05), aggregated at the phylum rank. | | | |
| --- | --- | --- | --- |
| Taxa† | Vegetation type‡  (tree station vs grass aisle) | Orchard^§^  (cider vs dessert) | Interaction^#^ |
| Ascomycota | 177; 103 | 447 | 54 |
| Basidiomycota | 25; 36 | 117 | 6 |
| Chytridiomycota | 6; 11 | 31 | 3 |
| Fungi | 101; 98 | 278 | 17 |
| Glomeromycota | 1; 15 | 18 | 1 |
| Rozellomycota | 3; 3 | 11 | 3 |
| Zygomycota | 5; 8 | 39 | 3 |
| Blastocladiomycota | 0; 1 | 1 | 0 |
| **Total** | **318; 275** | **942** | **87** |
| †Starting from the phylum rank—the lowest level of taxon with a UTAX confidence ≥ 0.65.  ‡The number of OTUs in each taxon which had higher abundance in tree station (before semicolon) and higher abundance in grass aisle samples (after semicolon).  ^§^The number of OTUs in each taxon which had different abundances between the two orchards.  ^#^The number of OTUs in each taxon which had different abundances between tree station compared to grass aisle samples at each orchard. | | | |

| **Table 6.** The number of bacterial OTUs with differential abundance (Benjamini-Hochberg corrected *p* ≤ 0.05), aggregated at the phylum rank. | | | |
| --- | --- | --- | --- |
| Taxa† | Vegetation type‡  (tree station vs grass aisle) | Orchard^§^  (cider vs dessert) | Interaction^#^ |
| Acidobacteria | 54; 61 | 445 | 82 |
| Actinobacteria | 19; 38 | 219 | 28 |
| Armatimonadetes | 3; 0 | 12 | 2 |
| Bacteria | 76; 73 | 603 | 63 |
| Bacteroidetes | 63; 40 | 248 | 45 |
| candidate division WPS-1 | 1; 7 | 34 | 1 |
| candidate division WPS-2 | 1; 8 | 20 | 2 |
| Candidatus Saccharibacteria | 11; 8 | 47 | 8 |
| Chlamydiae | 23; 2 | 54 | 11 |
| Chloroflexi | 7; 3 | 77 | 7 |
| Cyanobacteria/Chloroplast | 8; 0 | 3 | 4 |
| Euryarchaeota | 1; 0 | 3 | 0 |
| Firmicutes | 26; 5 | 68 | 19 |
| Gemmatimonadetes | 17; 1 | 61 | 11 |
| Hydrogenedentes | 1; 0 | 2 | 1 |
| Latescibacteria | 8; 5 | 35 | 7 |
| Nitrospirae | 5; 0 | 10 | 3 |
| Parcubacteria | 11; 2 | 62 | 13 |
| Planctomycetes | 10; 55 | 288 | 17 |
| Proteobacteria | 233; 130 | 911 | 177 |
| Verrucomicrobia | 22; 51 | 196 | 43 |
| Elusimicrobia | 0; 2 | 8 | 1 |
| Fibrobacteres | 0; 5 | 5 | 2 |
| Spirochaetes | 0; 3 | 5 | 2 |
| Tenericutes | 0; 3 | 3 | 2 |
| Aminicenantes | 0; 0 | 1 | 1 |
| BRC1 | 0; 0 | 6 | 0 |
| Ignavibacteriae | 0; 0 | 1 | 0 |
| Pacearchaeota | 0; 0 | 1 | 0 |
| Poribacteria | 0; 0 | 2 | 0 |
| Thaumarchaeota | 0; 0 | 1 | 1 |
| Woesearchaeota | 0; 0 | 3 | 0 |
| **Total** | **600; 502** | **3434** | **553** |
| †Starting from the phylum rank—the lowest level of taxon with a UTAX confidence ≥ 0.65.  ‡The number of OTUs in each taxon which had higher abundance in tree station (semicolon) and higher abundance in grass aisle samples (after semicolon).  ^§^The number of OTUs in each taxon which had different abundances between the two orchards.  ^#^The number of OTUs in each taxon which had different abundances between tree station compared to grass aisle samples at each orchard. | | | |

| **Table 7.** The number of fungal OTUs with differential abundance (Benjamini-Hochberg corrected *p*<=0.05), aggregated at the class rank. | | | |
| --- | --- | --- | --- |
| Taxa† | Vegetation type‡  (tree station vs grass aisle) | Orchard^§^  (cider vs dessert) | Interaction^#^ |
| Agaricomycetes | 10; 26 | 72 | 1 |
| Agaricostilbomycetes | 1; 0 | 0 | 0 |
| Ascomycota | 35; 32 | 120 | 8 |
| Basidiomycota | 4; 5 | 19 | 0 |
| Chytridiomycetes | 4; 3 | 12 | 0 |
| Chytridiomycota | 1; 8 | 18 | 3 |
| Dothideomycetes | 33; 13 | 56 | 10 |
| Eurotiomycetes | 21; 8 | 39 | 4 |
| Exobasidiomycetes | 2; 0 | 1 | 0 |
| Fungi | 101; 98 | 278 | 17 |
| Glomeromycota | 1; 7 | 9 | 0 |
| Lecanoromycetes | 2; 1 | 3 | 0 |
| Leotiomycetes | 26; 9 | 52 | 3 |
| Microbotryomycetes | 2; 4 | 8 | 2 |
| Monoblepharidomycetes | 1; 0 | 1 | 0 |
| Mortierellomycotina *Incertae sedis* | 3; 6 | 27 | 3 |
| Mucoromycotina *Incertae sedis* | 1; 1 | 1 | 0 |
| Orbiliomycetes | 7; 1 | 5 | 1 |
| Pezizomycetes | 14; 4 | 29 | 6 |
| Rozellomycota | 3; 3 | 11 | 3 |
| Saccharomycetes | 1; 3 | 4 | 1 |
| Sordariomycetes | 38; 31 | 130 | 19 |
| Tremellomycetes | 6; 1 | 15 | 3 |
| Zygomycota | 1; 1 | 10 | 0 |
| Blastocladiomycota | 0; 1 | 1 | 0 |
| Glomeromycetes | 0; 8 | 9 | 1 |
| Pezizomycotina Incertae sedis | 0; 1 | 6 | 1 |
| Geoglossomycetes | 0; 0 | 3 | 1 |
| Pucciniomycotina Incertae sedis | 0; 0 | 1 | 0 |
| Ustilaginomycetes | 0; 0 | 1 | 0 |
| Zygomycota Incertae sedis | 0; 0 | 1 | 0 |
| **Total** | **318; 275** | **942** | **87** |
| †Starting from the phylum rank—the lowest level of taxon with a UTAX confidence ≥ 0.65.  ‡The number of OTUs in each taxon which had higher abundance in tree station (before semicolon) and higher abundance in grass aisle samples (after semicolon).  ^§^The number of OTUs in each taxon which had different abundances between the two orchards.  ^#^The number of OTUs in each taxon which had different abundances between tree station compared to grass aisle samples at each orchard. | | | |

| **Table 8.** The number of bacterial OTUs with differential abundance (Benjamini-Hochberg corrected *p*<=0.05), aggregated at the class rank. | | | | | | |  |
| --- | --- | --- | --- | --- | --- | --- | --- |
| Taxa† | | Vegetation type‡  (tree station vs grass aisle) | | Orchard^§^  (cider vs dessert) | Interaction^#^ | |  |
| Acidobacteria | | 15; 5 | | 65 | | 9 | |
| Acidobacteria Group1 | | 2; 1 | | 30 | | 3 | |
| Acidobacteria Group10 | | 1; 6 | | 27 | | 3 | |
| Acidobacteria Group13 | | 2; 0 | | 4 | | 2 | |
| Acidobacteria Group15 | | 2; 0 | | 5 | | 1 | |
| Acidobacteria Group16 | | 4; 4 | | 33 | | 3 | |
| Acidobacteria Group17 | | 3; 4 | | 20 | | 4 | |
| Acidobacteria Group18 | | 1; 0 | | 2 | | 0 | |
| Acidobacteria Group2 | | 4; 0 | | 8 | | 2 | |
| Acidobacteria Group20 | | 1; 0 | | 1 | | 1 | |
| Acidobacteria Group22 | | 1; 3 | | 20 | | 4 | |
| Acidobacteria Group3 | | 6; 7 | | 39 | | 12 | |
| Acidobacteria Group4 | | 2; 5 | | 39 | | 6 | |
| Acidobacteria Group5 | | 2; 2 | | 10 | | 1 | |
| Acidobacteria Group6 | | 5; 19 | | 94 | | 23 | |
| Acidobacteria Group7 | | 1; 4 | | 19 | | 5 | |
| Actinobacteria | | 19; 37 | | 218 | | 28 | |
| Alphaproteobacteria | | 50; 18 | | 164 | | 36 | |
| Anaerolineae | | 2; 0 | | 14 | | 3 | |
| Armatimonadetes | | 1; 0 | | 10 | | 1 | |
| Armatimonadia | | 2; 0 | | 2 | | 1 | |
| Bacilli | | 2; 0 | | 25 | | 1 | |
| Bacteria(k) | | 76; 73 | | 603 | | 63 | |
| Bacteroidetes | | 14; 22 | | 94 | | 14 | |
| Bacteroidetes *incertae sedis* | | 5; 4 | | 25 | | 10 | |
| Bacteroidia | | 5; 0 | | 7 | | 2 | |
| Betaproteobacteria | | 33; 18 | | 102 | | 33 | |
| candidate division WPS-1 | | 1; 5 | | 30 | | 1 | |
| candidate division WPS-2 | | 1; 8 | | 20 | | 2 | |
| Candidatus Saccharibacteria | | 11; 8 | | 47 | | 8 | |
| Chlamydiae | | 6; 0 | | 14 | | 4 | |
| Chlamydiia | | 17; 2 | | 40 | | 7 | |
| Chloroflexi | | 5; 1 | | 38 | | 2 | |
| Chloroplast | | 8; 0 | | 2 | | 3 | |
| Clostridia | | 20; 1 | | 27 | | 14 | |
| Cytophagia | | 10; 2 | | 18 | | 5 | |
| Deltaproteobacteria | | 34; 48 | | 220 | | 34 | |
| Epsilonproteobacteria | | 1; 0 | | 1 | | 0 | |
| Euryarchaeota | | 1; 0 | | 2 | | 0 | |
| Firmicutes | | 2; 2 | | 12 | | 2 | |
| Flavobacteriia | | 8; 1 | | 16 | | 4 | |
| Gammaproteobacteria | | 92; 16 | | 238 | | 50 | |
| Gemmatimonadetes | | 17; 1 | | 61 | | 11 | |
| Holophagae | | 2; 1 | | 5 | | 2 | |
| Hydrogenedentes | | 1; 0 | | 2 | | 1 | |
| Latescibacteria | | 8; 5 | | 35 | | 7 | |
| Negativicutes | | 2; 0 | | 2 | | 1 | |
| Nitrospira | | 5; 0 | | 8 | | 2 | |
| Opitutae | | 2; 1 | | 8 | | 1 | |
| Parcubacteria | | 7; 1 | | 49 | | 9 | |
| Parcubacteria(p) | | 4; 1 | | 13 | | 4 | |
| Planctomycetes | | 1; 14 | | 51 | | 2 | |
| Planctomycetia | | 9; 38 | | 223 | | 15 | |
| Proteobacteria | | 23; 30 | | 184 | | 24 | |
| Spartobacteria | | 6; 18 | | 61 | | 14 | |
| Sphingobacteriia | | 21; 11 | | 88 | | 10 | |
| Subdivision3 | | 4; 24 | | 83 | | 22 | |
| Verrucomicrobia | | 3; 6 | | 26 | | 4 | |
| Verrucomicrobiae | | 7; 2 | | 18 | | 2 | |
| Caldilineae | | 0; 1 | | 10 | | 1 | |
| candidate division WPS-1(p) | | 0; 2 | | 4 | | 0 | |
| Elusimicrobia | | 0; 2 | | 7 | | 1 | |
| Erysipelotrichia | | 0; 2 | | 2 | | 1 | |
| Fibrobacteres | | 0, 5 | | 5 | | 2 | |
| Ktedonobacteria | | 0; 1 | | 13 | | 1 | |
| Mollicutes | | 0; 3 | | 3 | | 2 | |
| Phycisphaerae | | 0; 3 | | 14 | | 0 | |
| Spirochaetia | | 0; 3 | | 4 | | 2 | |
| Thermoleophilia | | 0; 1 | | 1 | | 0 | |
| Acidobacteria Group11 | | 0; 0 | | 3 | | 0 | |
| Acidobacteria Group12 | | 0; 0 | | 1 | | 0 | |
| Acidobacteria Group23 | | 0; 0 | | 1 | | 0 | |
| Acidobacteria Group25 | | 0; 0 | | 18 | | 1 | |
| Acidobacteria Group9 | | 0; 0 | | 1 | | 0 | |
| Aminicenantes(p) | | 0; 0 | | 1 | | 1 | |
| BRC1 | | 0; 0 | | 6 | | 0 | |
| Chloroflexia | | 0; 0 | | 2 | | 0 | |
| Cyanobacteria | | 0; 0 | | 1 | | 1 | |
| Endomicrobia | | 0; 0 | | 1 | | 0 | |
| Ignavibacteria | | 0; 0 | | 1 | | 0 | |
| Nitrospirae | | 0; 0 | | 2 | | 1 | |
| Oligoflexia | | 0; 0 | | 2 | | 0 | |
| Pacearchaeota(p) | | 0; 0 | | 1 | | 0 | |
| Poribacteria | | 0; 0 | | 2 | | 0 | |
| Spirochaetes | | 0; 0 | | 1 | | 0 | |
| Thaumarchaeota | | 0; 0 | | 1 | | 1 | |
| Thermoplasmata | | 0; 0 | | 1 | | 0 | |
| Woesearchaeota(p) | | 0; 0 | | 3 | | 0 | |
| **Total** | | **600; 502** | | **3,434** | | **553** | |
| †Starting from the phylum rank—the lowest level of taxon with a UTAX confidence ≥ 0.65.  ‡The number of OTUs in each taxon which had higher abundance in tree station (before semicolon) and higher abundance in grass aisle samples (after semicolon).  ^§^The number of OTUs in each taxon which had different abundances between the two orchards.  ^#^The number of OTUs in each taxon which had different abundances between tree station compared to grass aisle samples at each orchard. | | | | | |  |  |

# Experimental design, materials and methods

## Study design

Soil microbial communities were profiled in soil samples taken from two geographically and agronomically distinct apple orchards. Full information on the location and history of the two orchards is given in [1]. Within each orchard, soils were sampled from two vegetation types: former tree stations and the adjacent grassed aisles; which were divided into three blocks of ca. 20 m long, each with eight consecutive trees (i.e. eight pairs of tree and aisles samples). Three replicate soil cores (2.5 cm diameter, containing soil of 5 cm – 20 cm depth) were taken ca. 15 cm apart from each other at each type of sampling point (grass aisle and tree station).

## DNA extraction and sequencing

Total genomic DNA was isolated from 0.25 g of each soil sample using the protocol as described in [1]. Non-overlapping variable Internal Transcribed Spacer (ITS) regions of ITS1 and ITS2 were amplified using primers EkITS1F: (5’- CTT GGT CAT TTA GAG GAA GTA A -3’) and Ek28R : (5’- AT ATG CTT AAG TTC AGC GGG -3’). The V4 variable region of the 16S rRNA gene was amplified using primers F341 (5’– CCT ACG GGN GGC WGC AG–3‘) and R805 (5’– GGA CTA CHV GGG TAT CTA ATC C–3’). The two primer sets were modified at the 5’ end with adaptors, TCG TCG GCA GCG TCA GAT GTG TAT AAG AGA CAG – forward adaptor and GTC TCG TGG GCT CGG AGA TGT GTA TAA GAG ACA – reverse adaptor. Full PCR conditions and sequencing preparation are given in [1].

## Bioinformatic analysis of sequence reads

FASTQ sequences were processed to identify operational taxonomic units (OTUs) and calculate OTU abundances using UPARSE 9.0 OTU clustering pipeline [2]. Full details of all parameters used are given in [1].

### Assignment of taxonomic rank

The UTAX algorithm (<http://drive5.com/usearch/manual/tax_conf.html>) signed each OTU representative sequence to taxonomic ranks by alignment with the gene sequences in the reference databases ‘Unite V7’ (ITS) [3] and ‘RDP training set 15’ (16S) [4].

## Statistical analyses

All statistical analyses were carried out in R 3.2.0 [5]. OTU counts were normalised for library size using the median-of-ratios method implemented in DESeq2 [6-7]. The three samples taken from each sampling point were treated as analytical replicates and the data were pooled; for sampling points with less than three valid replicates (due to sequencing failures), the mean of available replicates was adjusted accordingly. OTUs with fewer than six normalised reads across all samples were excluded from further statistical analysis. All analyses were carried out separately for ITS and 16S data.

### Differential OTU abundance

DESeq2 was used to detect OTUs with differential relative abundances in relation to vegetation type, orchards and their interactions. DESeq2 implements an algorithm that automatically filters OTUs before differential abundance analysis based on several criteria, including variance in abundance across samples and overall abundance level. The fitted model was: spatial location within each orchard, vegetation type (grass vs tree), orchard (cider vs dessert), and the interaction between vegetation type and orchard. To correct for the false discovery rate associated with multiple testing, the Benjamini-Hochberg (BH) adjustment **[8]** was used within DESeq2. Statistical significance was determined at the 5 % level (BH adjusted).

# Acknowledgements

This research was funded by BBSRC, UK (grant number: BB/M01777X/1) and a consortium of industry partners: HEINEKEN UK Limited, Frank P. Matthews, Fruittree Rootstock Holland B.V., Vermeerderingstuinen Nederland, and A.C. Goatham & Son.

**References**

[1] G. Deakin, E.L. Tilston, J. Bennett, T. Passey, N. Harrison, F. Fernández, X. Xu

**Spatial structuring of soil microbial communities in commercial apple orchards**

Applied Soil Ecology (2018), in press

[2] R.C. Edgar

**UPARSE: highly accurate OTU sequences from microbial amplicon reads**

Nat. Methods, 10 (2013), pp. 996–998

[3] U. Koljalg, R.H. Nilsson, K. Abarenkov, L. Tedersoo, A.F. Taylor, M. Bahram, S.T. Bates, T.D. Bruns, J. Bengtsson-Palme, T.M. Callaghan, B. Douglas, T. Drenkhan, U. Eberhardt, M. Duenas, T. Grebenc, G.W. Griffith, M. Hartmann, P.M. Kirk, P. Kohout, E. Larsson, B.D. Lindahl, R. Lucking, M.P. Martin, P.B. Matheny, N.H. Nguyen, T. Niskanen, J. Oja, K.G. Peay, U. Peintner, M. Peterson, K. Poldmaa, L. Saag, I. Saar, A. Schussler, J.A. Scott, C. Senes, M.E., Smith, A. Suija, D.L. Taylor, M.T. Telleria, M. Weiss, K.H. Larsson

**Towards a unified paradigm for sequence-based identification of fungi**

Mol. Ecol., 22 (2013), pp. 5271–5277

[4] J.R. Cole, Q. Wang, J.A. Fish, B. Chai, D.M. McGarrell, Y. Sun, C.T. Brown, A. Porras‑Alfaro, C.R Kuske, J.M Tiedje

**Ribosomal Database Project: data and tools for high throughput rRNA analysis.**

Nucleic Acids Res., 42 (2014), pp. 633-642

[5] R Core Development Team

**R: A language and environment for statistical computing**

(2008)

[6] S. Anders, W. Huber

**Differential expression analysis for sequence count data**

Genome Biol., 11 (2010), pp. 106

[7] I.M. Love, W. Huber, S. Anders

**Moderated estimation of fold change and dispersion for RNA-seq data with DESeq2**

Genome Biol., 15 (2014), pp. 550

[8] Y. Benjamini, Y. Hochberg

**Controlling the false discovery rate: a practical and powderul approach to multiple**

**testing.**

J. R. Stat. Soc. Ser. B, 57 (1995), pp. 289–300
